# Integrating Large Language Models into Traffic Systems: Integration Levels, Capability Boundaries, and an Information-Theoretic Perspective

**DOI:** 10.3390/e28020211

**Published:** 2026-02-11

**Authors:** Wenwen Tu, Junfan Li, Feng Xiao, Xiaosa Wang, Yong Lu

**Affiliations:** 1 Engineering Training Center, Kunming University of Science and Technology, Kunming 650500, China; 20240246@kust.edu.cn; 2 School of Artificial Intelligence Industry, Kunming University of Science and Technology, Kunming 650500, China; 202310605132@stu.kust.edu.cn (J.L.); wangxiaosa@stu.kust.edu.cn (X.W.); luyong@stu.kust.edu.cn (Y.L.); 3 Business School, Sichuan University, Chengdu 610065, China

**Keywords:** large language models, intelligent traffic systems, information theory, uncertainty modeling, autonomous agents

## Abstract

Large language models (LLMs) are fundamentally transforming intelligent traffic systems by enabling semantic abstraction, probabilistic reasoning, and multimodal information fusion across heterogeneous data. This review examines existing research on LLM integration, ranging from data representation to autonomous agents, through an information-theoretic lens, conceptualizing LLMs as entropy-minimizing probabilistic systems that shape their capabilities in uncertainty modeling and semantic compression. We identify core integration patterns and analyze fundamental limitations arising from the inherent mismatch between discrete, entropy-driven LLM reasoning and the continuous, causal, and safety-critical nature of physical traffic environments. This reflects a deep structural tension rather than mere technical gaps. We delineate clear boundaries: LLMs are indispensable for managing high semantic entropy in tasks like contextual understanding and knowledge integration, whereas classical physics-based and optimization models remain essential in domains requiring ultra-low physical, temporal, and causal/normative entropy, such as real-time control and safety verification. Finally, we propose a forward-looking research agenda centered on hybrid intelligence architectures that bridge semantic information processing with physical system modeling for next-generation traffic systems.

## 1. Introduction

Complex real-world systems are fundamentally information-processing systems, in which uncertainty arises from incomplete observations, stochastic dynamics, and heterogeneous agent behaviors. Information theory and probability theory provide the foundational tools for modeling how information is represented, compressed, transmitted, and utilized for decision-making under uncertainty. In recent years, the emergence of large-scale probabilistic models has renewed interest in understanding how high-dimensional semantic information and uncertainty can be integrated into complex decision systems.

Traffic systems constitute a prototypical large-scale, open, and non-stationary information system. They are characterized by highly heterogeneous data modalities, strong spatiotemporal dependencies in their dynamic evolution, stochastic human behavior, and tightly coupled physical and safety-critical constraints [1]. Traffic intelligence therefore goes beyond perception and prediction, and inherently involves information fusion, uncertainty propagation, and coordinated decision-making across multiple temporal and spatial scales. Modeling and controlling such systems require not only accurate data-driven learning, but also principled mechanisms for managing uncertainty and aligning information with physical dynamics and operational constraints.

Large language models (LLMs) represent a recent paradigm shift in probabilistic modeling. Trained via entropy minimization objectives such as cross-entropy loss, LLMs learn high-dimensional conditional probability distributions over structured sequences, enabling powerful capabilities in semantic representation, information compression, and uncertainty-aware reasoning across heterogeneous modalities. These properties make LLMs attractive candidates for augmenting traffic systems, where diverse data sources and complex interactions demand flexible semantic integration and high-level reasoning beyond the scope of traditional task-specific models [2,3,4,5,6,7,8,9,10].

While traditional Deep Learning (DL) models have achieved notable success in specific traffic tasks, such as perception, short-term prediction, and pattern recognition, they exhibit inherent limitations in cross-modal semantic understanding, long-horizon causal reasoning, and flexible decision-making in open and evolving environments [11,12,13]. These limitations are closely related to their restricted ability to represent high-level semantic information and to explicitly manage uncertainty across heterogeneous data sources. In contrast, LLMs offer a unified probabilistic framework for semantic modeling and reasoning, opening new opportunities to address these challenges from an information-processing perspective.

Existing survey studies on the application of LLMs in traffic systems are predominantly organized around specific application scenarios, such as Autonomous Driving (AD), travel demand prediction, or traffic management [5,14,15,16,17,18]. Although this scenario-oriented organization helps illustrate the potential of LLMs across different traffic domains, it also introduces fragmentation in technical themes and analytical focus. Specifically, first, the same technical approaches (e.g., multimodal representation learning or knowledge-enhanced reasoning) may be applied across multiple scenarios, leading to repetitive coverage and fragmented technical discussions when categorized solely by application domain [2,19,20,21]. Second, the roles assumed by LLMs differ significantly across tasks, ranging from auxiliary representation enhancement to direct decision generation, yet existing surveys lack a unified analytical perspective to consistently and analytically compare these functional roles and their underlying information-processing mechanisms [22,23,24,25]. Furthermore, current reviews often fail to address a fundamental question from a probabilistic and information-theoretic standpoint: in what ways and at which system levels do LLMs participate in traffic task modeling, and how do uncertainty propagation, capability boundaries, and technical pathways vary across these levels [25,26,27].

To address these challenges, this paper adopts a four-level LLM integration perspective (representation, reasoning and prediction, planning and control, and autonomous agent integration) grounded in the depth of technical integration and the evolution of system autonomy. This hierarchy reflects different stages of information encoding, semantic compression, uncertainty transfer, and information–decision coupling within intelligent traffic systems. Conducting the literature review from this perspective offers three key advantages. First, it enables studies across diverse traffic scenarios to be compared in terms of representation strategies, reasoning mechanisms, and decision-making patterns, facilitating the identification of common technical structures and capability dependencies [2,15,22,28]. Second, the proposed classification implies an ascending capability progression in semantic reasoning and uncertainty handling, which allows for a discussion of which LLM capabilities have reached relative maturity in traffic applications and which remain constrained by fundamental limitations [25,26,27]. Third, by examining different levels of technical integration, it becomes possible to assess in which traffic tasks LLMs demonstrate clear advantages and in which contexts they remain unable to surpass traditional physics-based or model-driven approaches, as well as to analyze the underlying reasons for these differences [5,11]. The main contributions of this paper are summarized as follows:

- First, this paper analyzes the limitations of classical deep learning models in traffic applications from the perspectives of uncertainty modeling and information processing and distills the core modeling considerations and capability requirements at the intersection of LLMs and traffic optimization.
- Second, this paper conducts a narrative synthesis of LLM-based research in traffic representation, prediction and reasoning, planning and control, and autonomous agents under the proposed four-level integration perspective, revealing capability dependencies, recurring technical patterns, and differences in integration pathways across studies.
- Third, through comparative analysis, this paper discusses the applicability boundaries, failure modes, and appropriate modeling roles of LLMs in traffic tasks, and proposes corresponding directions for future research toward bridging entropy-based semantic reasoning and physical system modeling.

The remainder of this paper is organized as follows. Section 2 summarizes fundamental theories and modeling analysis of LLM applications in traffic systems. Section 3 introduces the four-level integration perspective and reviews research on intelligent traffic applications based on LLM technologies. Section 4 discusses the fundamental limitations and failure modes of LLM-enabled traffic systems. Section 5 outlines future research directions for LLM-based traffic applications. Section 6 concludes the paper.

## 2. Methodological Framework of the Narrative Review

This review employs a transparent, multi-source literature identification and synthesis methodology designed to capture both the rapidly evolving applied research and the essential theoretical foundations relevant to integrating Large Language Models (LLMs) into traffic systems.

### 2.1. Identification and Screening of Core Applied Literature

The primary objective was to assemble a broad and representative corpus of studies that implement or analyze LLMs within traffic and transportation contexts.

- Search Strategy and Sources (December 2025):

To ensure coverage of both archival publications and the latest advancements, our search spanned three complementary channels. We searched Scopus and Web of Science Core Collection for peer-reviewed journal articles and conference proceedings. We searched IEEE Xplore for relevant engineering literature and critically queried the arXiv preprint to capture cutting-edge work prior to formal publication. This inclusion is essential for a timely review of this fast-moving field. Google Scholar was used for backward/forward citation chaining of key papers to identify additional relevant studies.

- Exact Search Query:

The following Boolean query formed the basis, adapted per platform: (“large language model” OR LLM OR GPT OR “vision-language model”) AND (traffic OR transportation OR “autonomous driving” OR “traffic control” OR ITS).

- Screening Process and Inclusion Criteria:

The consolidated records were iteratively screened and curated through a two-stage process conducted by two authors to ensure relevance and analytical depth.

Stage 1: Title/Abstract Screening. Records were excluded if they: (i) did not involve an LLM/VLM in a substantive role; (ii) were not applied to a traffic/transportation task; (iii) were non-technical (e.g., editorials, news).

Stage 2: Full-Text Assessment. Retrieved articles were included only if they: (i) presented a concrete technical method or framework integrating LLMs with traffic data/control; (ii) provided sufficient detail to analyze the LLM’s functional role.

- Final Composition of the Core Applied Corpus:

This process resulted in a curated set of 98 representative studies that constitute the core analytical basis of this review. The composition of this corpus reflects the state of the field: it includes archival journal publications, conference papers, and pivotal arXiv preprints that represent the forefront of research.

### 2.2. Selection of Foundational and Contextual References

A separate set of references is cited to establish the necessary theoretical and comparative groundwork for our analysis. These were not retrieved via the applied literature search but were selected based on their seminal nature, authoritative status as surveys, or direct conceptual relevance to key themes in our discussion (e.g., information theory, model architectures, AI ethics).

These 14 contextual references include: Foundational neural architectures (e.g., the Transformer [29]). Authoritative surveys on classical deep learning models (CNNs [30], GNNs [31], RNNs [32]). General overviews of LLM capabilities and challenges [8,33]. Cross-domain studies illustrating key concepts relevant to our analysis (e.g., LLM-as-a-judge [6], code understanding [7], AI safety [34], ethical frameworks [35]).

### 2.3. Synthesis via the Four-Level Integration Framework

The 98 core applied studies were analyzed and categorized according to the four-level integration framework (representation, reasoning/prediction, planning/control, autonomous agents) presented in Section 3. The 14 contextual references provide the foundational concepts and vocabulary that inform the information-theoretic perspective and critical discussion throughout the paper.

### 2.4. Methodological Transparency and Limitations

This methodology is designed for transparency. The inclusion of arXiv preprints is explicitly justified by the field’s pace. We acknowledge limitations: the search is limited to English; the screening, though conducted with high inter-rater agreement, involves interpretative judgment; and the cut-off date means very recent works may be omitted. Entropy is not treated as a search keyword or inclusion criterion, as the majority of existing LLM-based traffic studies do not explicitly formulate their methods in information-theoretical terms. Instead, entropy is introduced as an analytical lens used to interpret uncertainty structures, capability boundaries, and failure modes across heterogeneous studies.

Accordingly, this review does not aim to provide an exhaustive or protocol-driven enumeration of all existing studies, but rather to develop a coherent analytical framework that explains capability patterns, structural limitations, and integration boundaries across representative LLM-based traffic research.

## 3. Fundamental Theories and Modeling Analysis of LLM Applications in Traffic

LLMs are probabilistic generative models capable of learning complex statistical regularities from large-scale data and performing sequence-based inference under uncertainty. Their core functionality lies in modeling high-dimensional conditional probability distributions through entropy minimization objectives, enabling semantic representation, information compression, and uncertainty-aware reasoning. Although LLMs are trained using cross-entropy loss functions, this training objective should not be interpreted as a direct operational guarantee of entropy minimization in downstream traffic tasks. Rather, it reflects optimization over symbolic sequence distributions during training, whose uncertainty-reduction properties may not transfer to tasks dominated by physical dynamics, causal intervention, or safety constraints.

This section first analyzes the fundamental limitations of classical deep learning models in traffic applications from the viewpoints of in-formation representation and uncertainty handling and then summarizes the theoretical and architectural foundations underlying the use of LLMs in the traffic domain. Starting from the evolution of core model architectures, this section discusses how architectural design and optimization strategies have progressively evolved to accommodate the in-formation complexity, multimodality, and dynamic uncertainty inherent in traffic systems.

### 3.1. Limitations of Classical Deep Learning Models in Traffic Applications

**Figure 1:**
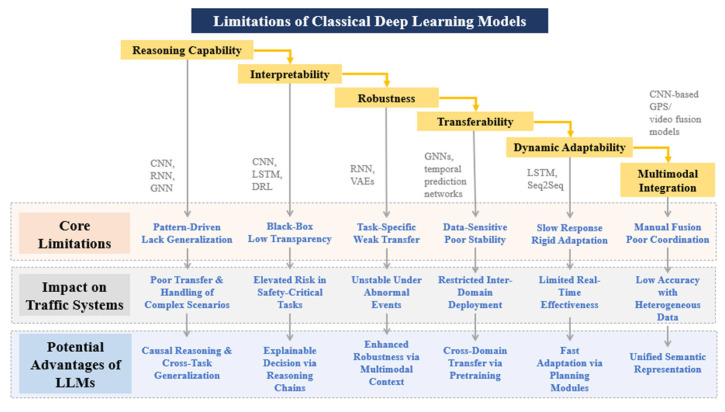
Limitations of Classical Deep Learning Models in Traffic Systems.

Although traditional deep learning approaches, such as Convolutional Neural Networks (CNNs), Recurrent Neural Networks (RNNs), Graph Neural Networks (GNNs), and Variational Autoencoders (VAEs), have achieved notable progress in traffic prediction, route planning, and signal optimization, they still exhibit significant limitations when applied to complex traffic system problems [30,31,32]. As summarized in Figure 1, these limitations can be interpreted from an information-theoretic and probabilistic perspective.

First, classical deep learning models are typically trained on data collected from specific cities, scenarios, or operational conditions, and rely heavily on large volumes of historical observations for pattern learning. This corresponds to learning conditional distributions under narrow and stationary data assumptions. When deployed in cross-task, cross-city, or cross-modal settings, distributional shifts and increased uncertainty lead to severe degradation in predictive performance and transferability. These models lack explicit mechanisms for uncertainty quantification and information generalization beyond the training distribution, which fundamentally limits their scalability and robustness.

Second, the interpretability and action transparency of traditional models remain limited. In safety-critical applications such as traffic safety analysis and autonomous driving, model outputs often lack explicit semantic explanations and causal attribution. This reflects the absence of structured information decomposition and uncertainty-aware reasoning within model architectures. As a result, abnormal behaviors or unexpected system responses are difficult to trace, diagnose, and correct, thereby increasing operational risk.

Third, classical deep learning models tend to be fragile in sparse, noisy, or long-tailed data environments. Low-frequency traffic events, sudden incidents, and missing sensor data introduce high uncertainty and information incompleteness. Since most traditional models implicitly assume dense and representative data distributions, they struggle to maintain stable inference when faced with elevated entropy in the input space, leading to unstable predictions and insufficient robustness in real-world deployments.

### 3.2. From Transformer Architectures to Multimodal and Agent-Based Systems

The technical foundation of LLMs originates from the Transformer architecture, which employs a self-attention mechanism to model long-range dependencies in sequential data. Self-attention enables adaptive information weighting and selective information aggregation across tokens, allowing models to dynamically allocate representational capacity to informative components of the input sequence. This architectural innovation effectively mitigates the information bottlenecks and vanishing dependency issues inherent in RNN-based models.

**Figure 2:**
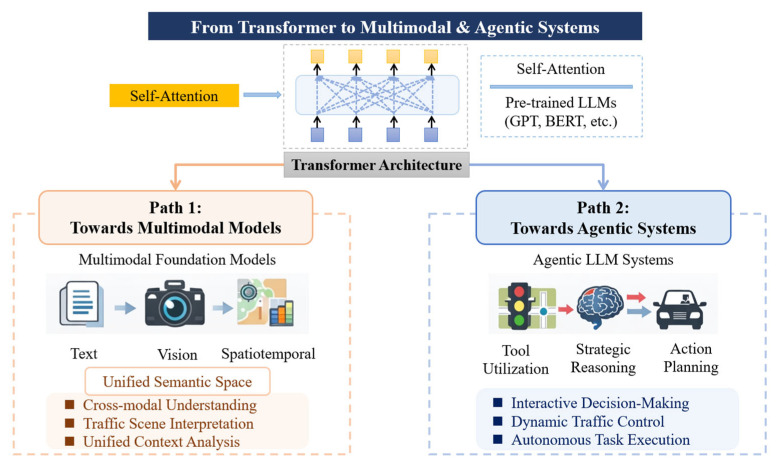
Schematic diagram of current technological development trends. The blue header denotes the common origin, while the gray block signifies the core Transformer architecture. Pre-trained models (e.g., GPT, BERT) are highlighted in the light blue dashed box. Solid arrows indicate the main technological progression toward two complementary paths: Path 1 (orange) illustrates the extension to multimodal models, integrating text, vision, and spatiotemporal data into a unified semantic space for traffic scene understanding. Path 2 (blue) depicts the evolution toward agentic systems, where LLMs interface with tools, reasoning modules, and planners to support interactive decision-making and dynamic control in traffic. Dashed arrows and outlines represent conceptual connections and modular integration, respectively, emphasizing that both directions are extensions of the same foundation.

Pre-trained language models such as Generative Pre-trained Transformer (GPT) and Bidirectional Encoder Representations from Transformers (BERT) acquire strong probabilistic priors for language understanding and generation through self-supervised learning on massive text corpora, using objectives such as Masked Language Modeling (MLM) and next-token prediction [29,33,36,37]. These objectives correspond to minimizing conditional entropy under incomplete information, thereby enabling models to learn rich semantic representations and uncertainty-aware inference mechanisms. However, purely text-based models remain insufficient to address the intrinsic multimodality and interactive decision requirements of traffic systems. Current technological developments can be broadly characterized by two major evolutionary directions, as illustrated in Figure 2.

The first direction is the progression toward multimodal foundation models. Traffic systems involve multiple information modalities, including textual data (e.g., incident reports and user queries), visual data (e.g., surveillance videos and sensor images), and spatiotemporal data (e.g., GPS trajectories and traffic flow measurements). Recent multimodal large models, such as GPT-4V and Gemini, map heterogeneous data sources into shared semantic spaces via aligned embedding representations or unified encoder–decoder architectures [2,4,20,38,39]. This multimodal alignment process can be interpreted as cross-modal information fusion, in which heterogeneous sources are jointly encoded to reduce semantic uncertainty and enable coherent reasoning across modalities. As a result, such models support complex tasks such as interpreting traffic scenes from visual inputs and inferring potential causes of congestion or incidents [19,40,41].

The second direction is the evolution toward agent-based systems. When LLMs are employed as the cognitive core of autonomous agents, their role extends beyond passive inference to active information-driven decision-making. These agents receive multimodal environmental inputs, invoke specialized tools or domain-specific models (e.g., routing algorithms and signal control modules), perform probabilistic reasoning and long-horizon planning, and ultimately generate control actions (e.g., adjusting variable message signs or providing negotiation strategies for Autonomous Vehicles (AV)) [7,15,23]. From an information-theoretic viewpoint, this evolution reflects a shift from static information modeling to closed-loop information–decision coupling, requiring LLMs to manage uncertainty over time and update beliefs based on feedback. Tool-use capabilities and feedback-driven planning enable LLMs to transition from content generation to interactive decision-making systems [42,43].

### 3.3. Core Capabilities and Architectural Adaptation of LLM-Enabled Traffic Systems

Effectively and reliably deploying general-purpose LLMs in safety-critical and dynamically complex traffic systems may not be achieved through prompt engineering alone. The dynamic, multimodal, and safety-sensitive nature of traffic systems requires LLMs to form a coherent cognitive stack spanning perception, semantic understanding, probabilistic reasoning, and decision generation. These capability requirements constitute a layered hierarchy, reflecting increasing levels of information abstraction and uncertainty handling. However, general-purpose LLMs are primarily optimized for semantic sequence modeling and lack explicit mechanisms for incorporating domain-specific constraints, physical dynamics, and risk-sensitive decision criteria.

**Figure 3:**
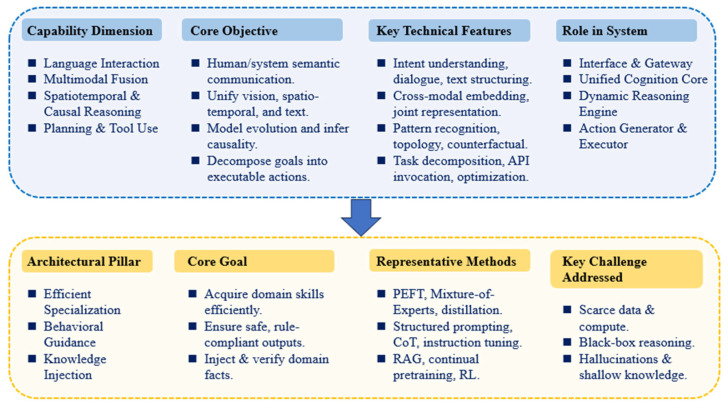
Core Capabilities for LLM-Enabled Traffic Systems and Key Architectural Adaptations of LLMs for Traffic Tasks.

Consequently, architectural adaptation is required to ensure reliable, controllable, and information-consistent behavior in traffic applications. This adaptation process includes domain-specific specialization to reduce semantic ambiguity, behavioral guidance mechanisms to constrain uncertainty-driven outputs, and deep integration of structured domain knowledge to align probabilistic reasoning with physical system dynamics. Representative architectural strategies supporting these objectives are summarized in Figure 3. Based on the capability and architectural analysis presented in this section, the remainder of this paper reviews how LLMs are integrated into traffic intelligence at different levels, namely representation, reasoning and prediction, planning and control, and autonomous agents.

### 3.4. LLM-Based Traffic Task Modeling

**Figure 4:**
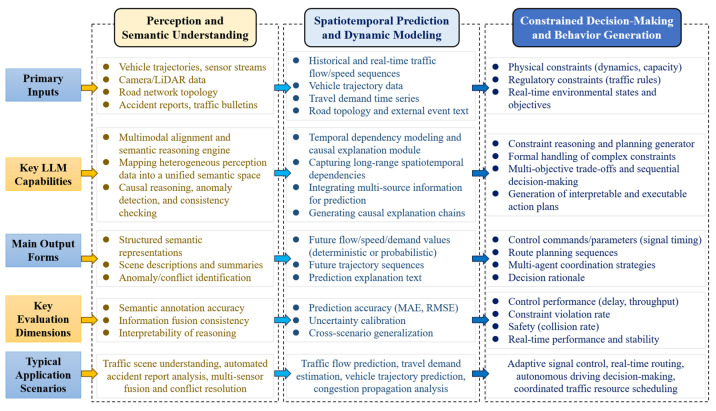
Core Dimensions, Capabilities, and Evaluation of LLM-Based Traffic Task Modeling.

LLM-based traffic task modeling leverages multimodal information fusion and probabilistic sequence modeling to transform heterogeneous traffic data, including text, images, trajectories, and road networks, into unified semantic representations, as shown in Figure 4. This process can be viewed as mapping high-entropy, heterogeneous observations into structured latent representations that support downstream reasoning and decision-making. A common modeling paradigm adopts a collaborative encoder–LLM architecture. Non-textual data are first processed by modality-specific encoders, such as visual encoders for images, temporal encoders for trajectories, and GNNs for road networks, to extract structured feature representations. These features are then aligned with the LLM input space via projection layers, enabling joint semantic reasoning. The LLM functions as the central probabilistic inference engine, integrating multimodal information through self-attention and performing mappings from semantic interpretation and uncertainty-aware reasoning to task-specific outputs. This process can be formally expressed as $f_{\theta }(X)\to Y,$ where X denotes the encoded multimodal inputs, Y represents task-specific outputs, and θ denotes model parameters.

### 3.5. Entropy Structures in Traffic Tasks and Implications for LLM Integration

To establish a precise analytical foundation, this review adopts a structured taxonomy of entropy in traffic system tasks, referring not to a single scalar quantity but to distinct forms of task-dependent uncertainty that arise at different layers of perception, reasoning, and interaction. These entropy structures are not intended as redefinitions of thermodynamic or Shannon entropy, but as analytically grounded characterizations of uncertainty that directly determine the suitability and limits of LLM integration.

#### 3.5.1. Three Hierarchical Levels of Entropy

We distinguish three hierarchical levels of entropy, within which all subsequent entropy-related terms used in this review are defined and situated.

- Representation-Level Entropy

Semantic entropy refers to uncertainty arising from ambiguity, incompleteness, or contextual dependence in symbolic, linguistic, or categorical representations. Typical examples include interpreting incident reports, extracting attributes from social media, explaining traffic regulations, or aligning textual and visual descriptions. This form of entropy resides in high-dimensional discrete symbol spaces and reflects uncertainty over meaning, labels, or relations. Owing to their autoregressive training objectives and token-level probabilistic modeling, LLMs are particularly effective at compressing and reducing semantic entropy.

Physical state entropy, in contrast, characterizes uncertainty over continuous traffic states governed by physical laws and kinematic constraints, such as vehicle position, velocity, headway, density, or signal phase evolution. This entropy propagates through deterministic or stochastic dynamic systems with strong temporal causality and safety-critical constraints. Regulating physical state entropy typically requires continuous representations, numerical solvers, and hard constraint enforcement—capabilities that are not intrinsically supported by discrete, token-based language model inference.

The representation-layer entropy mismatch denotes the structural asymmetry whereby reductions in semantic entropy achieved through discrete symbolic compression do not correspond to reductions in physical state entropy. This mismatch forms the theoretical basis for the limitations discussed in Section 5.1.1.

- Inference- and Prediction-Level Entropy

Correlation entropy describes uncertainty modeled through statistical dependence and conditional likelihoods learned from historical observations. Many LLM-based prediction frameworks effectively act as high-dimensional correlation entropy compressors, capturing regularities and co-occurrence patterns in spatiotemporal data. Such entropy reduction is valid under stationarity assumptions but does not encode causal mechanisms.

Causal uncertainty arises from incomplete knowledge of the underlying data-generating processes, including unobserved confounders, interventions, feedback loops, and structural changes. In traffic systems, causal uncertainty dominates under policy shifts, infrastructure changes, extreme events, or behavioral adaptation. Unlike correlation entropy, causal uncertainty cannot be resolved by probabilistic extrapolation alone and requires causal discovery, structural causal models, or counterfactual reasoning. The distinction between correlation entropy and causal uncertainty underpins the limitations analyzed in Section 5.1.2.

- Multi-Agent and System-Level Entropy

Strategic uncertainty (or epistemic uncertainty in multi-agent settings) reflects incomplete or asymmetric knowledge of other agents’ intentions, beliefs, and adaptive strategies. While LLMs can reason descriptively about such uncertainty, stable system-level outcomes often require explicit equilibrium modeling, coordination protocols, or mechanism design.

Social entropy refers to the amplification of uncertainty at the system level resulting from the interaction of multiple individually rational agents. Even when each agent locally minimizes its own uncertainty or expected cost, uncoordinated decision-making can increase aggregate variability, instability, or inefficiency—manifesting as synchronized route switching, secondary congestion, or cascading failures. Social entropy is therefore an emergent property of interaction rather than an attribute of any single agent, as discussed in Section 5.1.4.

Normative entropy captures uncertainty arising from value trade-offs, ethical constraints, and context-dependent objectives that cannot be reduced to a single scalar reward. In human–machine collaboration, institutional or legal entropy further emerges from ambiguity in responsibility attribution, accountability, and governance. These forms of entropy are not statistical in nature but reflect irreducible uncertainty over norms, rules, and authority, and they delimit the role of LLMs in socially embedded traffic systems.

For clarity, the entropy terms used in this review do not imply that a single universal metric is computed across tasks. Rather, each entropy structure admits different measurable proxies depending on modeling context. Semantic entropy may be operationalized through predictive entropy, token-level uncertainty, or output dispersion in language models. Physical state entropy corresponds to uncertainty over continuous states and may be characterized via state variance, reachable set bounds, or worst-case safety margins. Correlation entropy is typically reflected in predictive distribution spread under stationary assumptions, whereas causal uncertainty manifests as sensitivity to interventions or structural changes that cannot be captured by observational likelihoods alone. Social and normative entropy, in contrast, are not reducible to statistical entropy measures and instead reflect irreducible uncertainty in multi-agent coordination and governance. These entropy categories are not mutually exclusive; a single traffic task may simultaneously involve multiple entropy structures at different system levels.

#### 3.5.2. Implications for LLM Integration

The central argument of this review is that the effectiveness of LLM integration is determined not merely by data availability or model scale, but by the structural alignment between an LLM’s inference mechanism and the dominant entropy structure of the task. LLMs are powerful reducers of semantic and correlation entropy due to their alignment with symbolic and probabilistic modeling. However, their discreet, autoregressive generation is fundamentally mismatched for directly regulating physical state entropy, resolving causal uncertainty, or stabilizing emergent social entropy.

Importantly, “entropy” in this review does not denote a universally computable quantity. Different entropy structures may be characterized using predictive entropy, belief dispersion, causal uncertainty bounds, or worst-case guarantees, depending on context. The purpose of this taxonomy is not to conflate LLM training objectives with operational entropy minimization, but to clarify which uncertainties LLMs can effectively manage, which they cannot, and why hybrid architectures remain necessary. This framework provides the conceptual foundation for the four-level integration hierarchy and the capability boundaries examined in the remainder of the review. Throughout this review, describing LLMs as ‘entropy-minimizing’ refers strictly to their training objective in symbolic sequence modeling, and should not be interpreted as implying direct minimization of task-level physical, causal, or social entropy in downstream traffic systems.

## 4. Review of LLM-Based Intelligent Transportation Applications

### 4.1. LLM Integration Classification Perspective in Traffic Systems

**Figure 5:**
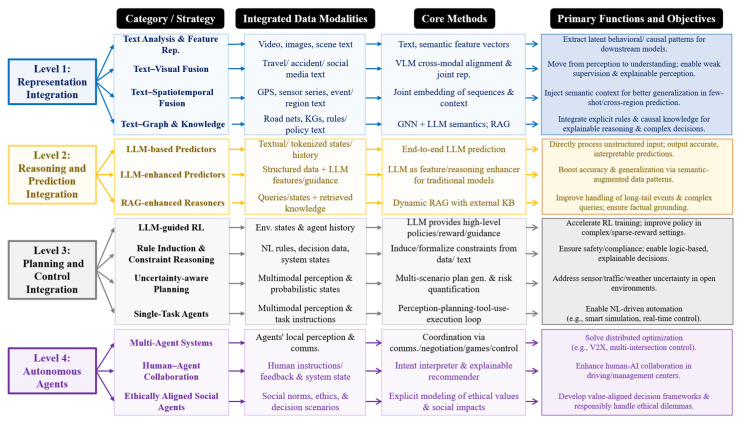
Four-Level LLM Integration Classification Perspective in Traffic Systems.

To organize existing studies, this paper proposes a four-level LLM integration classification perspective for traffic systems by jointly considering the functional architecture of traffic modeling and the hierarchical structure of information processing and decision-making. As illustrated in Figure 5, the proposed perspective consists of four dimensions: representation, reasoning and prediction, planning and control, and autonomous agent integration.

From an information-theoretic perspective, this classification reflects a progressive evolution in how uncertainty is represented, propagated, and reduced across different layers of traffic systems. Lower levels primarily focus on reducing observational entropy through multimodal data representation and semantic alignment, while higher levels emphasize uncertainty-aware inference, decision optimization, and closed-loop control under complex constraints. Rather than categorizing studies solely by application scenarios, this perspective highlights how LLMs participate at different depths of the traffic information-processing pipeline and how their roles evolve with increasing system autonomy.

Specifically, the proposed framework is structured along two coupled dimensions: the depth of technical integration and the degree of system autonomy. These dimensions together form a continuous evolutionary trajectory from low-level semantic representation to high-level autonomous decision-making. In practice, higher-level autonomous capabilities are fundamentally constrained by the reliability of lower-level representation quality and the robustness of reasoning mechanisms, since errors or uncertainty amplification at early stages can propagate through the system and degrade downstream decisions. Based on this classification perspective, the following sections present a review of existing LLM-based traffic studies.

It is important to emphasize that the proposed four-level integration perspective is not intended solely as a literature organization tool, but as an analytical framework for exposing differences in information coupling and system autonomy that are obscured by scenario-based classifications. Studies categorized under the same application label (for example, “autonomous driving”) may differ fundamentally in how LLMs are integrated. In some cases, LLMs are confined to auxiliary semantic roles such as annotation or rule interpretation, while in others they directly participate in planning or decision generation. Although these systems appear similar at the application level, they exhibit radically different risk profiles, uncertainty propagation mechanisms, and failure modes. Scenario-oriented taxonomies tend to obscure these distinctions, making it difficult to analyze capability boundaries and structural limitations. By contrast, the proposed integration levels explicitly reflect the depth of information coupling between LLMs and physical system dynamics, enabling a clearer assessment of when LLMs enhance system performance and when they introduce irreducible risk.

### 4.2. Research on Traffic Application of Representation Integrated

At the representation integration layer, LLMs are primarily employed to restructure heterogeneous traffic data into semantically coherent forms, thereby reducing representational uncertainty and enabling information fusion across modalities. It focuses on transforming high-dimensional, noisy, and weakly structured inputs into compressed semantic representations that preserve task-relevant information while discarding redundancy.

#### 4.2.1. Traffic Application Research Based on Pure Text Analysis

Studies based on pure text analysis constitute a foundational and highly extensible semantic entry point for traffic applications. In this line of research, LLMs function as semantic representation learners and high-level concept abstractors, transforming unstructured textual data, such as incident reports, policy documents, user feedback, or operational logs, into unified latent representations that support downstream decision-making, evaluation, and governance tasks [36,44,45]. As shown in Table 1, these approaches leverage the probabilistic language modeling capability of LLMs to implicitly capture complex traffic semantics and contextual dependencies, even under weak supervision or limited labeled data. By learning conditional distributions over traffic-related textual sequences, LLMs effectively perform semantic compression, reducing uncertainty while retaining interpretable intermediate concepts. Such representations can then be reused or transferred across tasks, enabling flexible adaptation and improved generalization.

The following subsections and tables (Table 1, Table 2, Table 3, Table 4, Table 5, Table 6, Table 7, Table 8, Table 9, Table 10, Table 11, Table 12, Table 13 and Table 14) organize representative studies according to our four-level framework, focusing on their core technical approach, the functional role of the LLM, and the key problem addressed. It should be noted that these studies differ substantially in experimental scale, evaluation depth, and deployment maturity. Works that do not report system-level performance metrics, baseline comparisons, or long-term operational validation are treated in this review as proof-of-concept or exploratory prototypes rather than mature deployment-ready systems. The field of LLM-integrated traffic systems is rapidly evolving, with a high proportion of exploratory proofs-of-concept, simulation-based validations, and short-horizon experimental demonstrations. Many studies, particularly those proposing novel architectures or leveraging foundation models in new ways, prioritize demonstrating functional feasibility and potential capability over comprehensive system-level benchmarking against established baselines, long-term operational stability, or real-world deployment metrics. To mitigate heterogeneity in reporting practices across the surveyed literature, this review focuses on comparative analysis at the level of integration depth, functional role, and uncertainty handling mechanism, rather than on direct performance comparison. Quantitative metrics, datasets, and baselines are reported where available; however, their absence in many studies reflects the early exploratory nature of LLM integration in traffic systems rather than an omission by this review. Readers are therefore encouraged to interpret the tables as a mapping of capability patterns and failure modes, rather than as a benchmark-style comparison.

#### 4.2.2. Traffic Application Research Based on Text–Visual Fusion

In traffic text–visual fusion studies, LLMs primarily serve as high-level semantic understanding and reasoning interfaces. These models map complex traffic scenes—initially encoded as low-level visual, trajectory, or sensor features—into language-based semantic spaces that are jointly interpretable by humans and machine systems [2,3,4,19,20,38,39,40,41,46,47,48,49]. This fusion process can be viewed as cross-modal entropy alignment, in which heterogeneous modalities with distinct statistical structures are projected into a shared semantic space with reduced ambiguity. LLMs enable this alignment by acting as probabilistic decoders that associate visual patterns with linguistic concepts, thereby facilitating reasoning, explanation, and decision support. The core technical pathways and functional roles of LLMs in representative studies are summarized in Table 2.

**Table 2:** Representative Studies of LLM-Based Text–Visual Fusion in Traffic Systems.

Classification	Ref.	Traffic Task	Visual/Language Input	Fusion Strategy/LLM’s Role	Problem Addressed
Semantic Alignment & Verification	[2]	Scene dataset construction	Scene images. VQA text	VQA-driven alignment/Semantic representation support	Lack of traffic-domain semantics in general MLLMs
	[46]	Driving scene segmentation	Scene images. Language-supervised embeddings	Language-assisted alignment/Semantic constraint module	Purely visual segmentation lacks semantic constraints
	[47]	Efficient 3D data annotation	Point clouds/images. Consistency prompts	Language-guided verification/Semantic consistency verifier	Semantic drift in 3D annotations
Deep Cross-Modal Fusion	[20]	EV charging demand prediction	Satellite imagery. Structured prompts	Spatial semantic alignment/Urban functional reasoner	Remote sensing lacks usage semantics
	[4]	3D environment perception for AD	Camera & LiDAR. Cross-modal features	Feature-level attention (MoE)/Semantic-space fusion enhancer	Heterogeneous sensor alignment
	[48]	Efficient ITS data management	Multimodal sensor data. Compression prompts	Knowledge-driven reconstruction/Data compression enhancer	Low efficiency in multimodal data storage/transmission
Interactive Task & QA	[49]	Vehicle monitoring & interaction	Vehicle images. Detection-driven QA prompts	Task pipeline coordination/Interactive query controller	Single-function models lack semantic interaction
Deep Semantic Understanding	[38]	Holistic scene understanding	Scene images. Scene descriptions	Shared embedding space/Scene reasoning module	Vision models lack holistic & relational semantics
	[39]	Scene generation, QA, explanation	Traffic sign images. Traffic instructions	Domain-adaptive MLLM/Generation & QA agent	General MLLMs lack traffic-specific knowledge
	[40]	Traffic accident analysis	Traffic videos. Explanation queries	Video–language reasoning/Causal explanation generator	Fragmented “detect-then-analyze” pipelines
Real-Time Dynamic Analysis	[41]	Accident prediction, AD support	Real-time scene visuals. Contextual instructions	Real-time VLM fusion/Context enhancer & decision assistant	Traditional models ignore real-time visual/text context
	[19]	Human–machine interaction in AD	Driving scene images. NL instructions	Cross-modal attention/Instruction grounding executor	Grounding complex language in dynamic scenes
Unified Multimodal Model	[3]	Multi-sensor fusion, planning	Images, videos. (Implicit) time-series	LLM architecture/Explanation & decision analyzer	Inefficiency of multiple specialized models

#### 4.2.3. Traffic Application Research Based on Text–Spatiotemporal Fusion

Existing studies on text–spatiotemporal fusion typically reformulate traffic spatiotemporal dynamics into sequential or embedding-based representations that can be processed by LLMs, enabling them to act as unified spatiotemporal dependency modelers [50,51,52,53,54,55,56,57,58,59,60,61]. This approach allows LLMs to capture long-range temporal correlations and complex spatial interactions that are difficult to model using conventional architecture. This integration transforms high-dimensional spatiotemporal uncertainty into structured semantic sequences, enabling LLMs to model conditional dependencies across time and space within a single generative framework. As shown in Table 3, such methods often exhibit enhanced generalization performance under small-sample, cross-region, and non-stationary conditions.

**Table 3:** Representative Studies of LLM-Based Text–Spatiotemporal Fusion for Traffic Prediction.

Classification	Ref.	Spatiotemporal Data	Fusion Strategy/LLM’s Role	Key Advantage
Semantic Feature Enhancement	[50]	Bicycle flow sequences	Text descriptions, LLM embeddings; as semantic enhancer	Improves prediction under special events by integrating contextual text.
Architectural Improvement & Fusion	[51]	Road network sequences	CNN + GCN embeddings; as few-shot predictor	Mitigates the problem of historical data scarcity.
	[52]	Spatiotemporal flow data	Fusion layer embeddings; as spatiotemporal interaction enhancer	Strengthens the learning of temporal-spatial relationships.
	[53]	Flow with exogenous factors	Multi-source attention; as fluctuation modeler	(Metro passenger flow prediction)Integrates complex exogenous influencing factors.
	[54]	Road network sequences	Condensed Spatial Prompting; as frozen predictor	Highly efficient; compresses graph info into prompts.
	[55]	Spatiotemporal sequences	GAT + unified embeddings; as cooperative modeler	Decoupled design leverages graph networks (space) & LLMs (time).
	[56]	Large-scale road network flow	Lightweight generative model; as edge predictor	Reduces central cloud pressure via edge deployment.
spacetime tokenizer, partial frozen attention	[57]	Urban flow data	Spatiotemporal tokenizer; as reasoning module	Enables strong zero-/few-shot transfer capability.
	[58]	Location-based features	Partially frozen attention; as dependency capturer	Robust in low-data regimes; captures global dependencies.
Robust & Probabilistic Modeling	[59]	Multimodal system data	Denoising diffusion; as structure restorer	Robust to input noise and missing data.
	[60]	Road network sequences	Probabilistic modeling; as adaptive predictor	Adaptable and interpretable under data distribution shifts.
Global Unified Modeling	[61]	Charging series & spatial data	Multi-source embeddings; as global modeler	(EV charging prediction) Unifies heterogeneous spatiotemporal and contextual data.

#### 4.2.4. Traffic Application Research Based on Text–Graph and Knowledge Integration

**Figure 6:**
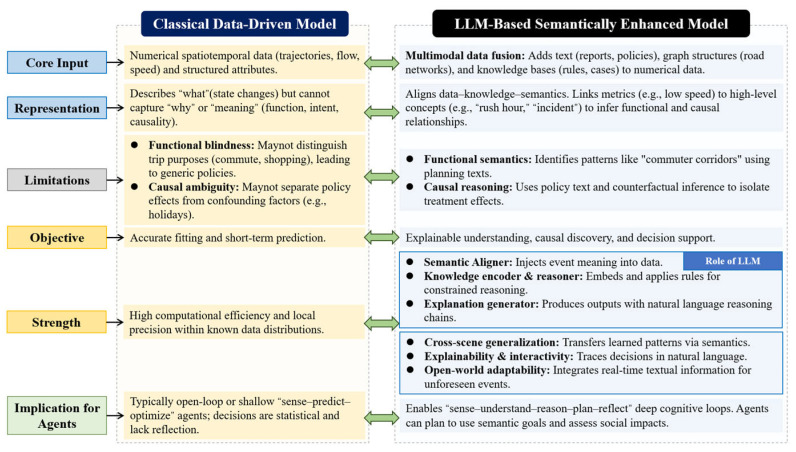
Comparison of Data-Driven and Semantically Enhanced Transportation Modeling.

In complex real-world traffic systems, relying solely on continuous numerical spatiotemporal data is often insufficient to capture higher-level system logic, such as intent, functionality, and causality. As summarized in Table 4 and Figure 6, recent studies have begun to incorporate graph structures and explicit knowledge into LLM-centered traffic modeling frameworks, positioning LLMs as semantic interpreters, knowledge aligners, and logical reasoners [62,63,64,65,66,67,68,69]. This integration enables semantic-level alignment between natural language knowledge descriptions and numerical traffic data, effectively constraining the hypothesis space of the model and reducing reasoning uncertainty [66,68]. By internalizing rules, constraints, and historical patterns, LLMs support interpretable inference over complex traffic phenomena, such as chained system responses induced by control policies or infrastructure changes [62,67]. Compared with purely data-driven approaches, this paradigm shifts traffic modeling from correlation-dominated learning toward function-aware and causality-informed reasoning [63,64,65,69].

**Table 4:** Representative Studies of LLM-Based Text-Graph & Knowledge Integration.

Ref.	Graph/Knowledge	LLM Access Method	LLM’s Core Role	Key Problem Addressed	Methodological Edge	Task
[62]	Traffic accident KG	RAG + KG construction	KG builder & QA enhancer	Manual KG construction; poor interactivity	Semi-auto extraction, RAG reduces hallucination	Accident Q&A & causal analysis
[63]	Cross-domain EV KG	Not directly used	Target for LLM integration	Scattered EV ecosystem knowledge	Provides structured KB for LLM apps	EV decision support
[64]	Traffic accident KG	Not directly used	Classic KG framework	Complex multi-dimensional data	auto KG construction	Accident visualization
[65]	Ship collision KG	NLP + ontology	Baseline for LLM methods	Lengthy reports; inefficient extraction	Ontology-based semi-auto extraction	Maritime accident analysis
[66]	Regional demand graph	Geo-semantic embedding	Cross-city encoder	Poor graph model generalization	Transferable semantic priors	Delivery demand prediction
[67]	Road behavior KG	RAG + KG reasoning	Retriever & explainer	Black-box, uninterpretable predictions	Explainable prediction	Pedestrian & lane-change prediction
[68]	Trajectory semantics	Multi-source encoding	Travel reasoner	DL struggles with travel semantics	Explicit semantic fusion	Pedestrian mode identification
[69]	Traffic element hierarchy	Hierarchical CoT	Scene analyzer & generator	Uncontrollable simulation	CoT + Frenet for control	Controllable AD scenario simulation

#### 4.2.5. Summary of Research on Representation Integration

**Figure 7:**
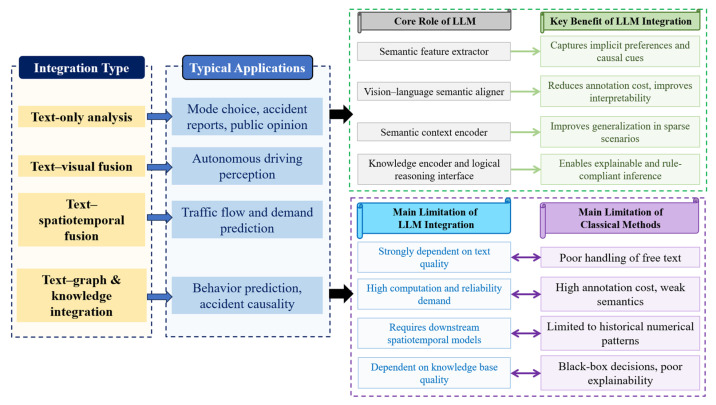
System Roles and Capability Boundaries of LLMs at Representation Integration. The yellow blocks denote different representation-level integration types of LLMs, while the light blue blocks indicate their corresponding traffic applications. Solid blue arrows represent functional mapping from integration type to application domain. The green dashed box summarizes the core roles and benefits of LLMs at this level, with green arrows indicating capability contributions enabled by semantic modeling. The blue dashed box highlights the main limitations of LLM integration, whereas the purple dashed box presents limitations of traditional statistical methods. Bidirectional dashed arrows indicate complementary and contrasting weaknesses between LLM-based and classical approaches. Dashed boundaries emphasize conceptual grouping rather than strict architectural separation.

LLMs at the representation integration layer primarily function as unified semantic alignment mechanisms for multi-source heterogeneous traffic information. They are embedded at the front-end or data processing stages of traffic intelligent systems to enable cross-modal semantic fusion, structured knowledge injection, and high-level feature abstraction. It reduces input uncertainty and improves information efficiency, thereby providing context-aware and knowledge-informed representations for downstream prediction, planning, and control tasks. Figure 7 summarizes application types, system roles, and capability boundaries at this layer, highlighting how different integration pathways correspond to distinct traffic scenarios and semantic gains.

### 4.3. Research on Traffic Applications at the Reasoning and Prediction Integration

While representation integration focuses on structuring information, the reasoning and prediction integration layer concerns how uncertainty is inferred, propagated, and resolved to support forecasting and decision-making.

#### 4.3.1. Research on LLM-Based Traffic Prediction

In contrast to the representation-level integration, research at the reasoning and prediction layer fundamentally re-conceptualizes traffic prediction as a semantic information decoding and generation problem. This approach addresses the inherent aleatoric and epistemic uncertainty within traffic systems by mapping heterogeneous inputs into a unified representation space, where LLMs can perform probabilistic inference to mitigate predictive entropy [20,28,59,70,71,72,73,74,75]. Unlike conventional numerical predictors that often rely on deterministic regression, LLM-based prediction models exhibit enhanced generalization capacity under conditions of high information sparsity or domain shift. This advantage stems from their ability to leverage semantic priors learned from vast corpora, effectively reducing the out-of-distribution entropy that typically degrades traditional models. As summarized in Table 5, this capability has been demonstrated across diverse tasks including electric vehicle (EV) charging demand forecasting, traffic flow prediction, and driving behavior intent inference [20,28,59,72,73,74,75].

Furthermore, to address the critical challenge of epistemic uncertainty in black-box predictions, recent studies have introduced information-theoretically motivated interpretability techniques. Methods such as Chain-of-Thought (CoT) reasoning and textualized predictions serve to increase mutual information between model inputs and outputs, effectively exposing intermediate inference logic and providing transparent mechanisms for uncertainty calibration and entropy analysis [28,70,73,74].

**Table 5:** Comparative Analysis of LLM-Based Traffic Prediction Applications.

Classification	Ref.	Task	LLM Role	Input Form/Prediction Output	Technical Feature	Main Contribution
Zero-shot/Few-shot Prompt Reasoning	[70]	mode choice	Zero-shot explainer	Task & attribute prompts/Mode + explanation	Zero-shot; CoT reasoning	Matches classic models without training data.
	[71]	Next location	Zero-shot predictor	Textualized history/Location + reasoning	Zero-shot; geo-knowledge	proof of zero-shot mobility prediction; strong in cold-start.
Multimodal and Hybrid Enhancement	[72]	EV charging demand	Direct predictor	Textualized spatiotemporal data/Demand sequence	End-to-end text-to-text	Reduces feature engineering; good generalization.
	[28]	Lane-change prediction	Explainable predictor	NL scene prompts/Intent & trajectory	Fine-tuning; CoT	LLM for lane-change prediction; explainable.
	[73]	Traffic flow prediction	Explainable predictor	NL data descriptions/Future flow	Instruction tuning	Builds explainable flow prediction model.
	[20]	EV charging demand	Multimodal predictor	Image + text prompts/Station demand	Vision-semantic learning	Robust cross-scene prediction via visual semantics.
	[74]	Transit demand	Hybrid system	Aligned flow/OD data/Value + explanation	Modular; prompt tuning	Flexible prediction with event-aware explanations.
	[75]	CAV misbehavior detection	Adaptive detector	Textualized V2X messages/Authenticity (class)	Hybrid fine-tuning	Unified detection of forged CAV signals/motions.
Dedicated Arch.	[59]	Multimodal sys.	Robust predictor	Heterogeneous sequences/Flow & demand values	Diffusion; ST-LLM	Unified framework for noisy multimodal data.

#### 4.3.2. Research on LLM-Enhanced Traffic Prediction

Another line of research positions LLMs as auxiliary reasoning modules rather than direct predictors. In this mode, LLMs enhance traditional prediction models through contextual understanding, feature reconstruction, reasoning guidance, or explanation generation [2,11,12,13,76]. Unlike representation integration, LLMs here intervene directly in the inference stage, helping manage uncertainty arising from incomplete observations, anomalous patterns, or rule-intensive scenarios, as summarized in Table 6.

**Table 6:** Representative Studies on LLM-Enhanced Prediction in Traffic Systems.

Ref.	Traffic Task	Role of LLM	Enhancement Mechanism	Main Contribution
[11]	Traffic flow prediction	Responsibility-aware predictor	Multimodal textification and causal reasoning	Introduces reliability- and responsibility-oriented prediction
[76]	Trajectory prediction	Semantic interaction enhancer	Implicit semantic modeling via pretrained LLM	Demonstrates robustness in few-shot settings
[12]	Accident severity inference	Context modeling and explanation	Table-to-text conversion and interpretable reasoning	Achieves accurate and explainable severity prediction
[13]	Flow and demand prediction	Reasoning-guided feature reordering	Prompt engineering and multi-step reasoning	Improves generalization under small samples
[2]	Traffic scene understanding	Semantic infrastructure	Multimodal QA dataset construction	Strengthens LLM reasoning foundation for prediction

#### 4.3.3. Research on RAG-Enhanced Traffic Reasoning

Many critical traffic prediction and decision problems depend on structured background knowledge, such as regulations, behavioral norms, and operational rules [67,77,78]. LLMs relying solely on implicit parametric knowledge are vulnerable to hallucination and inconsistency in such settings [26,79,80]. To address this issue, Retrieval-Augmented Generation (RAG) frameworks explicitly incorporate external knowledge sources during inference, effectively constraining the posterior distribution of model outputs. By performing real-time retrieval and fusion of domain knowledge, RAG-based methods reduce reasoning entropy and improve accuracy, interpretability, and robustness [26,67,77,79]. Alternative strategies inject domain expertise via parameter-efficient fine-tuning methods such as LoRA [78,80]. Representative studies are summarized in Table 7.

**Table 7:** Representative Studies on RAG-Enhanced Traffic Reasoning.

Ref.	Scenario	Retrieved Knowledge	Reasoning Role of LLM	Main Contribution
[67]	Behavior prediction	KG, behavioral relations	Causal reasoning with retrieval	Achieves explainable participant behavior prediction
[77]	Public transit services	Operational DB and policies	Constraint-aware reasoning hub	Extends RAG to rule-intensive service reasoning
[79]	Autonomous disengagement analysis	Historical report corpus	Pattern discovery and diagnosis	Enables large-scale root cause analysis
[80]	Risk assessment	Scenario-specific knowledge base	Causal and progressive reasoning	Reduces hallucination via external constraints
[78]	Fault diagnosis	Parameterized expert knowledge	Expert-level reasoning	Demonstrates parameterized RAG via LoRA
[26]	Traffic signal control	Historical decision memory	Experience-based coordination	Integrates RAG with Actor–Critic control

#### 4.3.4. Comprehensive Analysis of the Reasoning and Prediction Integration

**Figure 8:**
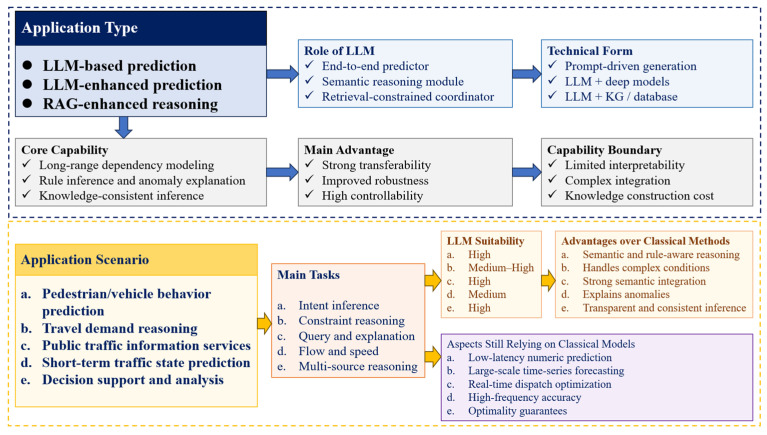
Application Types, System Roles, and Capability Boundaries at the Reasoning and Prediction Integration.

Overall, LLMs are embedded into predictive inference pipelines, where their primary function shifts from fitting pure data to the probabilistic management and reduction in systemic entropy. As illustrated in Figure 8, LLMs actively participate in uncertainty decomposition, contextual information fusion, and entropy-constrained inference across multi-source information streams.

LLM-based predictive approaches, which attempt end-to-end forecasting of traffic states or demand, inherently grapple with high-dimensional predictive entropy. While they demonstrate strong generalization, their reliance on implicit representations often obscures the distinction between aleatoric (inherent data) uncertainty and epistemic (model) uncertainty, making them vulnerable to entropy explosion under data distribution shifts and real-time constraints. In contrast, LLM-enhanced prediction methods strategically deploy LLMs as semantic reasoning modules to target specific information bottlenecks. They compensate for traditional models’ weaknesses in contextual understanding and anomaly handling by explicitly modeling and reducing semantic and causal uncertainties derived from unstructured data or complex rules.

The integration of RAG with traffic reasoning represents a principled information-theoretic intervention. By constraining the LLM’s generative entropy with curated knowledge from structured Knowledge Graphs (KGs) or real-time databases, RAG frameworks minimize hallucinatory divergence and maximize the mutual information between the model’s output and verifiable domain facts. This directly enhances reliability and interpretability in scenarios like behavioral prediction or causal query resolution. However, this architectural sophistication introduces new layers of complexity entropy, raising challenges in system design, dynamic knowledge governance, and maintaining inference latency within information-theoretic efficiency bounds.

When analyzed through the lens of entropy across different traffic scenarios, LLM-augmented techniques show superior capability in domains characterized by high semantic entropy and strategic uncertainty, such as user intent prediction and rule-intensive scenario reasoning. Yet, in high-frequency, low-latency numerical forecasting tasks where the dominant uncertainty is well-quantified aleatoric noise, the additional computational and informational complexity introduced by LLMs may not justify marginal gains, leaving classical models more entropy-efficient.

### 4.4. Research on Traffic Applications at Planning and Control Integration

At the planning and control integration layer, the role of LLMs fundamentally shifts from information representation toward entropy-aware decision regulation. It addresses how uncertainty, once encoded and propagated through perception and prediction, is actively managed, constrained, and transformed into executable actions under physical, safety, and operational constraints. In traffic systems, planning and control can be interpreted as a process of entropy allocation and reduction under uncertainty: future traffic states are inherently stochastic, control actions influence probability distributions rather than deterministic outcomes, and decision quality depends on how uncertainty is structured rather than eliminated. LLM-enabled planning and control mechanisms therefore operate not merely as optimization tools, but as semantic and probabilistic regulators that reshape the information landscape of decision-making.

#### 4.4.1. Research on LLM-Guided Reinforcement Learning (RL) Traffic Applications

LLM-guided Reinforcement Learning (LLM-guided RL) represents a class of approaches in which LLMs are directly embedded into the policy learning process. Unlike conventional RL, which relies on manually engineered rewards and extensive trial-and-error sampling, LLM-guided RL introduces semantic priors and high-level probabilistic structure into the learning loop.

Classical RL suffers from high reward entropy and sparse feedback, particularly in complex traffic environments where objectives are delayed, multi-dimensional, and difficult to formalize. LLM-guided RL alleviates these limitations by injecting structured information (derived from language, rules, or expert knowledge) into the policy optimization process, thereby reducing uncertainty in policy gradients and accelerating convergence. This decision–execution decomposition assigns LLMs to operate at the level of high-entropy semantic reasoning, while RL handles low-entropy numerical optimization. Such separation enables more stable learning in open and non-stationary traffic environments. Representative studies are summarized in Table 8. Existing approaches can be categorized into three main types [22,24,25,26,81,82,83,84,85,86,87,88,89,90].

- LLM-guided RL (Reward-centric)

The reward-centric paradigm focuses on using LLMs or Vision–Language Models (VLMs) to generate or reshape reward functions [81,82]. Reward shaping can be interpreted as a process of information densification, where sparse and delayed feedback is transformed into more informative and temporally distributed signals. Language-driven reward shaping typically follows three forms: (A) LLMs generate semantic rewards by aligning natural language objectives with environmental observations, thereby reducing ambiguity in reward attribution [81]; (B) LLMs iteratively revise reward structures through contextual reasoning, effectively adapting the information content of rewards based on learning feedback [82]; (C) In hierarchical RL, LLMs specify high-level intentions that constrain lower-level reward landscapes, indirectly regulating policy entropy through goal abstraction.

- High-level Planning in Hierarchical RL

Beyond reward-centric designs, an increasing number of studies positions LLMs as high-level planners within hierarchical RL architectures. In these systems, LLMs operate on abstract state representations and long-horizon objectives, generating meta-actions or strategic plans that guide lower-level policies. This corresponds to separating epistemic uncertainty from control uncertainty. LLMs reason over high-level semantic uncertainty, while RL optimizes actions within a constrained and lower-entropy action space. Representative forms include: (A) LLM-based strategic planners that translate complex traffic contexts into meta-actions [22,24,83]; (B) Decision refinement modules where RL produces candidate actions and LLMs reduce ambiguity through semantic reasoning and explanation [26,84,85]; (C) LLM-centric decision agents, where LLMs directly generate actions and classical controllers serve as stabilizing components [25,86].

- Priors and Constraints for RL Exploration

Concurrently, the third category leverages LLMs as generators of informational priors and semantic constraints. These approaches strategically use LLMs not for direct action selection, but to reduce the entropy of the search space and encode high-level domain knowledge, thereby providing a structured, information-rich guidance for the learning process. This category can be further delineated into three principal mechanisms: (A) Semantic State-Action Space Re-mapping. LLMs act as information translators, bridging the semantic gap between low-level sensorimotor data and high-level task abstractions. By re-mapping raw state observations or control variables into semantically meaningful representations, LLMs effectively compress the informational complexity of the environment, enabling RL agents to operate in a lower-entropy, more interpretable action space [87]. (B) Knowledge-Guided Exploration for Entropy Reduction. LLMs provide informed priors that constrain the initial policy distribution, dramatically reducing the exploratory entropy and associated sample complexity during early learning phases. This targeted injection of domain knowledge accelerates convergence and inherently biases exploration toward safer and more promising regions of the state-action space, mitigating the risks of random, high-entropy exploration in safety-critical settings [88]. (C) Strategic Coordination Guidance in Multi-Agent Systems. In Multi-Agent Reinforcement Learning (MARL) environments characterized by exponentially escalating strategic entropy, LLMs serve as high-level coordinators. By reasoning over global states and shared objectives, they generate cooperative protocols or suggest high-value joint policies. This function reduces the misalignment and coordination entropy among agents, guiding the decentralized policy search toward socially preferable equilibria [89,90].

**Table 8:** Representative Studies of LLM-Guided RL in Traffic Planning and Control.

Classification	Subclass	Ref.	Scenario	LLM Role	Guidance Mechanism	Key Contribution
LLM-guided RL (Reward-centric)	Semantic reward generation from language goals	[81]	AD	Reward generator (with VLM)	Language goals, semantic rewards	Contrastive language rewards reduce collisions & improve generalization.
	Automatic reward construction and evolution	[82]	Bus holding control	Reward function evolver	LLM generates/optimizes reward functions	Automatic reward evolution enhances control stability & robustness.
High-level Planning in Hierarchical RL	LLM-based High-level Planners	[22]	AD	High-level planner	Long-term goals & meta-action guidance	Improves generalization in complex scenes & decision explainability.
		[83]	Traffic signal control	Scene interpreter & decision generator	Perception, semantic reasoning, action generation	Vision-LLM joint control: LLM reasons for direct action, RL as fallback.
		[24]	Complex urban signal control	Reasoning decision center	Tool-augmented LLM, zero-shot reasoning	Hybrid framework for zero-shot adaptation & robust control in complex scenarios.
	LLM-enhanced Decision Refinement Modules	[84]	On-ramp merging control	Decision optimizer & enhancer	RL decisions to LLM CoT refinement	LLM refines/generalizes RL decisions for efficiency & safety in congestion.
		[85]	Highway AD	Explainable trajectory predictor	RL meta-action + state, trajectory generation	Cascaded framework: RL, meta-actions, LLM, safe trajectories, controller, execution.
		[26]	Displaced left-turn control	AC optimizer	Dual-agent: LLM Actor (decisions), Critic (RAG/memory)	GPTTC: LLM-based AC with RAG for adaptive control, reducing delay/stops.
	LLM-centric Decision Agents	[25]	Traffic signal control	Core decision agent	State → LLM (LightGPT), direct action	LLMLight: Specialized LLM agent for direct, efficient, explainable control.
		[86]	Adaptive signal control	Knowledge-accumulating agent	Zero-shot CoT & GCA with interactive learning	Generalist LLM agent (GCA) learns interactively, generates adaptive phases.
Priors and Constraints for RL Exploration	Semantic Action or State Space Re-mapping	[87]	Traffic signal control (Sim-to-Real)	Dynamics interpreter & action transformer	State, text, LLM reasoning, action remapping	PromptGAT: Uses LLM to understand dynamics & bridge sim-to-real gap via prompt-driven action transformation.
	Knowledge-guided Exploration and Initialization	[88]	Vehicle powertrain/energy	Prior knowledge coordinator	Initialization & exploration constraints	Reduces sample complexity, speeds convergence, improves energy efficiency.
	Coordination and Policy Search Guidance	[89]	Network traffic optimization	Analysis & prediction module	Data insights, RL action guidance	LLM-RL co-optimization: LLM predicts bottlenecks for RL signal/route guidance.
		[90]	Multi-intersection signal control	Multi-agent collaborator	LLM as reasoning component in MARL	LLM-enhanced MARL integrates Transformer, improves multi-agent collaboration.

**Figure 9:**
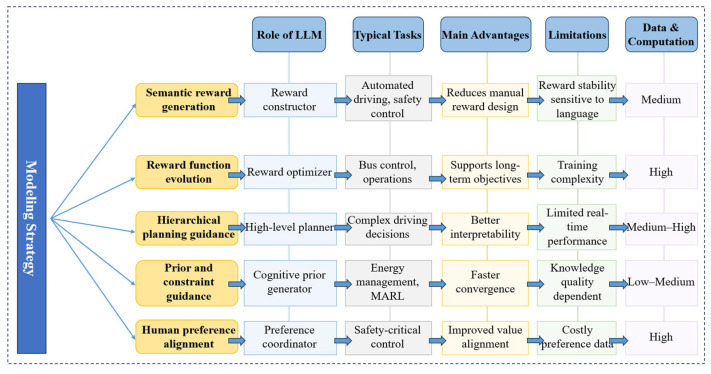
Modeling Strategies, Applicable Tasks, and Capability Boundaries of LLM-Guided RL.

The modeling strategies, applicable tasks, and fundamental capability boundaries of this LLM-guided RL mode are summarized in Figure 9.

Among the surveyed studies, the direct, reward-centric guidance of RL by LLMs remains less prevalent in traffic applications. The prevailing trend emphasizes their role in high-level strategic abstraction and structured exploration guidance. This shift underscores a consensus that the core value of LLMs lies not in optimizing low-level control loops, such as a domain with stringent requirements for temporal determinism and low computational entropy, but in providing the semantic scaffolding and informational constraints necessary for efficient and aligned RL. While these advances enhance goal alignment and generalization, they concurrently introduce new challenges pertaining to the consistency of semantic guidance, the stability of generated priors, and the computational overhead of maintaining a hybrid architecture.

#### 4.4.2. Research on Traffic Applications of LLM-Based Rule Induction and Constraint Reasoning

In traffic planning and control, effective decision-making must navigate a complex interplay between optimization-driven continuous actions and discrete, high-entropy rule systems derived from regulations, operational norms, and experiential knowledge. Traditional methods, which rely on manually curated rule sets or explicit constraint modeling within optimization frameworks, are inherently limited in their capacity to capture the latent, dynamically evolving, and semantically rich informational constraints that govern real-world traffic behavior [1,5,12,21,91,92,93,94,95]. To address this fundamental limitation, recent research investigates the application of LLMs for automated rule induction and probabilistic constraint reasoning. These studies, summarized in Table 9, leverage the unique ability of LLMs to reduce the semantic entropy of unstructured inputs, such as regulatory texts, incident reports, or behavioral narratives, and transform them into actionable decision rules or formalized constraints [12,91]. Beyond mere extraction, LLMs serve as semantic intermediaries that guide classical optimizers or simulation models by generating or interpreting constraints, thereby injecting high-level, context-aware informational structure into low-level numerical optimization processes [5,91,92,94,95].

This research direction focuses on three information-theoretic objectives: the induction of implicit knowledge structures from complex data [12,91]; the formalization of latent constraints into a machine-actionable representation; and the orchestrated coordination between learned, probabilistic rule systems and deterministic, classical models of traffic flow and control [91,92,95]. The integration aims to create hybrid systems where LLMs manage the high-dimensional uncertainty and semantic complexity of real-world rules, while traditional models ensure physical fidelity and computational tractability.

**Table 9:** Representative Studies of LLM-Based Rule Induction & Constraint Reasoning in Traffic Planning and Control.

Ref.	Application Scenario	LLM Role	Rule & Constraint Approach	Key Contribution
[91]	Urban delivery optimization	Implicit rule inducer	Behavior to language; learns human constraints + TSP	Shows LLMs learn complex rules to enhance classical optimization.
[5]	Urban traffic management	Task/rule coordinator	NL rule understanding; multi-model reasoning	TrafficGPT: LLM-foundation model collaboration for interactive decision.
[92]	AD rule formalization	Rule-to-logic translator	NL rules to CoT to MTL generation	TR2MTL: Auto-translates rules to formal MTL, enabling scalable verification.
[93]	Green wave control	Strategy generator/analyzer	NL-driven interactive strategy generation	Explores LLM for interactive green wave strategy design.
[94]	Intersection conflict mgmt.	Real-time conflict resolver	Zero-shot reasoning based on rules	Validates LLMs for real-time rule-based conflict prediction & resolution.
[12]	Traffic safety analysis	Domain-expert rule inducer	Structured narratives; fine-tuning; interpretability	CrashSage: LLM as explainable safety engine for causal rule induction.
[1]	General ITS	Unified reasoning core	Dual (physical + semantic) state-space theory	Theoretical framework unifying physical dynamics & semantic rules.
[21]	End-to-end AD	Behavior semanticizer	Intent-based control + NL explanation (VICS)	DriveLLM-V: Translates control to NL (VICS) to explain driving rules.
[95]	Signal control optimization	Feasible config generator	LLM generates constraint-satisfying phase timings	Validates off-the-shelf LLMs for auto-generating high-quality signal configs.

#### 4.4.3. Research on Traffic Applications of LLM-Based Uncertainty-Aware Planning

Real-world traffic environments are characterized by inherent informational entropy arising from stochastic participant behaviors, non-stationary environmental dynamics, and imperfect perception systems. Traditional deterministic planning methods or those dependent on single-point predictions fail to address the multimodal uncertainty distribution and long-tail risks inherent in such complex systems [27,42,96,97,98,99,100,101]. The integration of LLMs into planning frameworks introduces a mode shift toward explicit entropy management and information-theoretic decision-making. As summarized in Table 10, LLMs contribute by learning latent behavioral distributions from historical data and representing future states as probabilistic forecasts. This capability transforms planning inputs from deterministic trajectories to entropy-characterized probability distributions, thereby reducing over-reliance on a presumed single optimal future. The core value lies in providing distributional inputs that enable risk-sensitive planning under uncertainty, where decisions are optimized not for a single outcome but across the entropy spectrum of possible futures [98,99]. Consequently, the role of LLMs is evolving from passive uncertainty representation to active entropy-constrained optimization and uncertainty governance [27,42,96,99].

Early research primarily leveraged LLMs to enhance the accuracy and diversity of multimodal probabilistic predictions, effectively capturing a broader range of potential state evolutions [98]. Recent advancements, however, position LLMs as central components within closed-loop decision architectures. These systems perform iterative information gain maximization through integrated modules for dynamic risk (entropy) quantification, context-aware memory retrieval, and reflective reasoning. This allows for the continuous refinement of plans by actively seeking to minimize executional entropy while satisfying safety and performance constraints, marking a transition toward information-theoretically grounded, adaptive decision systems [27,42,96,99].

**Table 10:** Representative Studies of LLM-Based Uncertainty Planning.

Ref.	Scenario	Role of LLM	Uncertainty Modeling	Key Contribution
[96]	Urban signal control (complex scenarios)	Strategy generator & HMI coordinator	ACP strategy library; LLM generates novel strategies with human feedback	Proposes LLM-driven control paradigm with autonomous/feedback/manual modes for uncertainty.
[97]	AD scenario testing	Scenario & environment generator	Text, LLM, dynamic synthesis of virtual environments	Explores LLMs for generating diverse, uncertain driving scenarios for edge-case testing.
[98]	Trajectory prediction for AD & IoT	Semantic encoder & pattern extractor	LLM + spatiotemporal encoding + Normalizing Flows	First LLM integration into probabilistic trajectory prediction for multimodal uncertainty.
[99]	AD (high-risk & long-tail)	Risk reasoning & decision optimizer	Risk quantification + memory retrieval + reflective learning	SafeDrive: Modular system for context-aware safe decisions in uncertain, high-risk scenarios.
[42]	Traffic simulation & policy testing	Interactive planning agent	NL translation of policy goals; agent handles uncertainty	AgentSUMO: Agentic framework for interactive scenario generation & policy experimentation.
[100]	AD testing (pedestrian)	Pedestrian behavior generator	LLM configures diverse, context-sensitive behaviors via prompting	LLM-enhanced traffic editing to inject complex pedestrian behaviors for realistic AV testing.
[101]	AD scenario generation	Scenario augmentation agent	Language-guided, fine-grained scene augmentation	AGENTS-LLM: Generates OOD, challenging scenarios for planner evaluation.
[27]	Urban intersection control	Real-time traffic controller	LLM reasoning integrates data, resolves conflicts via CoT	Proposes LLMs as direct real-time controllers for dynamic traffic uncertainty.

#### 4.4.4. Summary and Analysis of Planning and Control Integration

**Figure 10:**
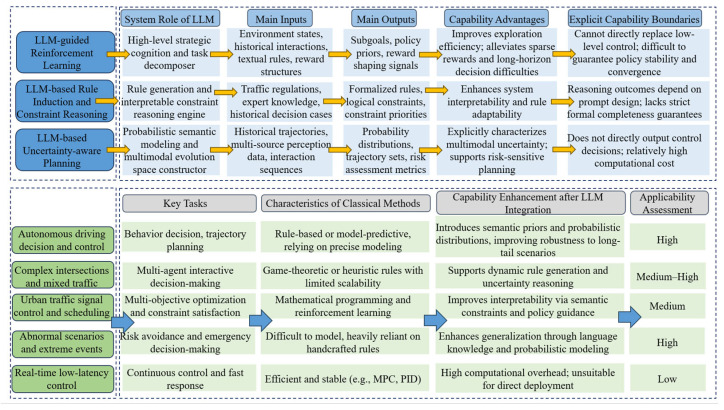
Application Types, System Roles, and Capability Boundaries of LLMs at Planning and Control Integration.

As summarized in Figure 10, the application of LLMs across planning and control paradigms can be understood through their distinct roles in managing and reducing specific types of systemic entropy.

In RL-guided scenarios, LLMs primarily function as high-level cognitive entropy regulators. They provide structured semantic priors and abstract task decompositions to the reinforcement learning agent, effectively constraining the exploration entropy of the low-level policy search. This strategic guidance transforms an unstructured, high-entropy exploration problem into a more focused and information-efficient learning process.

Within the domain of rule induction and constraint reasoning, LLMs act as unified semantic inference engines. They process explicit knowledge and implicit experiential data to extract and formalize latent rule structures, thereby reducing the semantic and regulatory entropy inherent in unstructured traffic governance. This capability allows them to dynamically generate or refine interpretable constraints, bridging the gap between high-level human-understandable rules and low-level, machine-actionable optimization frameworks.

Finally, in uncertainty-aware planning frameworks, LLMs are leveraged as probabilistic semantic modelers. Their core function shifts to characterizing and quantifying the multimodal entropy of future state evolutions. By transforming raw observations into structured probability distributions, they supply entropy-quantified inputs for downstream risk-sensitive decision-making, enabling planners to explicitly reason about and hedge against various forms of predictive and strategic uncertainty.

### 4.5. Research on Traffic Applications at the Autonomous Agent

At the autonomous agent layer, LLMs become self-contained probabilistic decision systems that integrate perception, reasoning, memory, and action. This layer represents the highest degree of autonomy and the most complex form of entropy management in traffic systems.

#### 4.5.1. Research on Traffic Applications of Single-Task Autonomous Agents

In the development of single-task autonomous agents, LLMs are primarily utilized as decision-making executors, tasked with autonomously completing specific objectives, such as route choice or facility planning, within defined constraints. A critical research focus lies in understanding how these agents process and manage the informational entropy inherent in traffic environments to achieve reliable task execution. This subsection reviews progress in this domain through an information-theoretical lens, with existing studies categorized into three distinct groups based on their approach to uncertainty handling and complexity management, as summarized in Table 11 [7,15,23,25,27,42,43,102,103,104,105,106].

The first category comprises traffic information processing and analysis agents. These systems employ domain-specific fine-tuning, real-time database querying, and multi-source information fusion to autonomously mine, interpret, and generate insights from large volumes of structured and unstructured data. Their core function is semantic entropy reduction: transforming heterogeneous, noisy data streams, such as accident reports or social media feeds, into actionable, low-entropy decision support for real-time monitoring and safety consultation [102,103].

The second category focuses on traffic behavior and system simulation agents. By equipping LLMs with structured memory, personalized attributes, and perception–decision modules, these studies enable the simulation of traveler decision-making in activities like route selection or parking search. The key contribution here is the modeling of behavioral entropy, capturing the complexity and variability of human preferences while providing interpretable, low-entropy explanations for simulated actions [15,104,105].

The third category involves traffic system control and simulation interaction agents. Through deep integration with external tools and simulation environments, along with techniques like Chain-of-Thought prompting and real-time reasoning, LLMs in this category perform strategic entropy regulation. They interpret high-level objectives, autonomously plan task sequences, and generate control strategies aimed at reducing systemic uncertainty and optimizing operational efficiency [7,42,43,106].

The fourth category advances into dedicated architecture and physical control. This line of research designs specialized LLM-based architectures or tightly couples LLMs with control systems to enable direct, real-time decision-making for tasks such as intersection management and traffic signal optimization. The emphasis here is on minimizing executional entropy under strict latency and safety constraints, ensuring that high-level decisions translate into reliable, low-variance physical control actions [23,25,27].

Overall, current research demonstrates that LLM-based single-task agents can effectively address a spectrum of traffic challenges by targeting specific entropy sources, whether semantic, behavioral, strategic, or operational. However, significant barriers remain, particularly in managing the trade-offs between decision complexity and real-time performance, ensuring safety under distributional shift and uncertainty, and achieving computational efficiency at scale. These challenges underscore the need for continued innovation in entropy-aware agent design for robust real-world deployment.

**Table 11:** Representative Studies of LLM-based Single-Task Autonomous Agents.

Classification	Ref.	Agent Task	Role of LLM	Key Technical Design	Main Contribution	Limitations
Zero-shot/Few-shot Reasoning	[102]	Accident monitoring	Info extraction agent	Social media data; multi-task learning	First LLM multi-task learning on accident tweets	Data quality dependent; not full system view
	[103]	Cycling info support	Service agent	Geospatial data; prompting; orchestration	Reproducible method for personalized safety info	Needs real-world validation
Task Reformulation & Instruction Tuning	[104]	Traffic safety consultation	Expert agent	LLaMA fine-tuning; safety standards alignment	First domain-specific LLM for safety; professional responses	Text-only; no real-time system integration
	[15]	Real-time traffic monitoring	Analysis agent	GPT-4 + DB; auto SQL; CoT; multi-agent	NL to complex query mapping; lowers analysis barrier	No direct physical control
	[105]	Parking search	Behavior sim agent	Persona; uncertain decision contexts	Simulates risk preferences & utility trade-offs	Lacks real behavioral data validation
Hybrid Enhancement & Modular Architecture	[7]	Traffic task scheduling	General agent	NL instruction parsing; tool invocation	Open-TI: end-to-end autonomous task execution	High complexity; toolchain dependent; costly
	[43]	Daily route choice	Traveler agent	Memory; persona; retrieval; LLM reasoning	Human-like route switching with explanations	Less stable than equilibrium models; high compute
	[106]	Parking planning	Planning agent	Structured prompts; modular chains	Flexible planning tool for AV/HDV transition	Manual workflow; non-real-time
	[42]	Simulation & policy test	Simulation agent	Goal understanding; task planning; SUMO tools	NL-driven simulation setup; lowers barrier	SUMO dependent; scenario validity unverified
Dedicated Architectures & Physical Control	[23]	Traveler behavior sim	Conceptual agent	Structured modules; activity-based alignment	LLM as rich agent in ABM; new demand modeling path	Conceptual; scalability/efficiency unproven
	[27]	Intersection control	Control agent	CoT; fine-tuning; conflict resolution	LLM as real-time controller under uncertainty	Preliminary; needs safety/scalability validation
	[25]	Signal control	Core controller	Custom LightGPT; state, reasoning, action	Efficient, generalizable, interpretable control	Specialized model; safety in edge cases unclear

#### 4.5.2. Research on Traffic Applications of Multi-Agent Collaborative Systems

Research on multi-agent collaborative systems investigates how LLMs can manage the escalated complexity and emergent entropy inherent in coordinating multiple agents within dynamic traffic environments [14,15,26,90,101,107,108]. The core challenge shifts from single-agent task completion to the systemic regulation of interaction entropy, aiming to achieve coordinated objectives like safety and efficiency through enhanced communication, intent alignment, and strategic consistency. As summarized in Table 12, LLMs in these frameworks serve as central information fusion and entropy arbitration hubs. They utilize abstract, language-driven reasoning to mitigate the coordination entropy that often plagues MARL approaches, specifically addressing limitations in generalization, interpretability, and modeling complex cooperative equilibria. The LLM’s role is to reduce misalignment uncertainty between agents and enforce global constraints, thereby transforming a high-entropy multi-agent system into a more predictable and cooperative ensemble.

**Table 12:** Summary of LLM-based Traffic Applications in Multi-Agent Collaborative Systems.

Multi-Agent Type	Ref.	Role of LLM	Collaboration Mechanism Design	Main Contributions	Limitations
Interactive Vehicle/Entity Systems	[107]	Regional & global collaborative reasoning engine	Hybrid: Individual RL + LLM coordination + RAG	Enhances safety and human-likeness in multi-vehicle merging.	Complex architecture; high cost.
	[90]	Multi-agent collaborative reasoning enhancer	LLM reasoning embedded within MAPPO framework.	Improves coordination efficiency of signal control.	Increases training complexity.
Functionally Divided Dual-Agent Systems	[108]	Trajectory generator & constraint evaluator	Generator–discriminator dual-channel collaboration.	Improves trajectory safety and controllability.	Relies on fixed architecture; lacks adaptive learning.
	[26]	Action optimizer (Actor) & policy evaluator (Critic)	Dual-agent Actor-Critic framework with LLMs.	Achieves adaptive control under near-saturated traffic.	Limited to single intersections; stability unverified.
Hierarchical & Conceptual Systems	[14]	Core component & potential coordinator of future ITS.	Conceptual framework based on multimodal learning.	Provides visionary insights into LLM-centered ITS.	Purely conceptual; lacks empirical validation.
	[101]	Scenario augmentation task agents.	Language-guided multi-agent collaboration.	Enables generation of challenging traffic scenarios.	Scenario physical validity depends on LLM.
Collaborative Task-Handling Systems	[15]	Traffic management & analysis agent.	Multi-agent collaboration for complex query handling.	Improves task completion in traffic monitoring.	Internal collaboration, not true multi-agent cooperation.

#### 4.5.3. Research on Traffic Applications of Human–Machine Collaborative Agents

In human–machine collaborative systems, LLMs as semantic mediators designed to bridge the cognitive and information-theoretic gap between human users and complex traffic data or models [34,77,109,110]. Unlike fully autonomous agents, these systems are architected for continuous informational exchange, keeping humans within the decision loop. The LLM’s function extends beyond retrieval or generation to actively modulate the entropy of human–machine interaction. This involves clarifying ambiguous human intent (reducing semantic input entropy), providing causal explanations for system recommendations (increasing decision transparency and mutual information), and coordinating interactive tasks [77,110]. As detailed in Table 13, such agents enhance comprehension efficiency and decision consistency for end-users in applications like travel consultation. The evolution in this domain reflects a progression from merely improving interaction efficiency toward achieving deeper cognitive alignment and shared situational awareness, effectively minimizing the joint entropy of the collaborative human–machine system.

**Table 13:** Summary of LLM-based Traffic Applications in Human–Machine Collaborative Agents.

Ref.	Scenario	Human Role	LLM’s Role	Collaboration Mechanism	Contributions	Limitations
[109]	Aviation communication training	Pilot (Trainee)	Professional language trainer	Keyword-driven scenario & dialogue generation	Enables low-cost communication training	Language-only; lacks control integration
[77]	Public transport services	Passenger, Dispatcher	Conversational agent & data interpreter	Natural language interaction with data query/feedback	Enhances information accessibility	Dependent on high-quality structured data
[110]	Travel recommendation	Traveler	Interactive recommender & explainer	Feedback-driven iterative recommendation	Facilitates serendipitous travel discovery	Limited to small-scale empirical tests
[34]	Human–machine co-driving	Driver	Empathetic cognitive partner	Multimodal emotion recognition & adaptive interaction	ethical–emotional governance framework	Conceptual; lacks quantitative validation

#### 4.5.4. Research on Traffic Applications of Ethically Aligned Social Agents

The evolution of autonomous traffic systems toward social acceptability necessitates agents that can navigate not only functional but also ethical and normative entropy [34,35,111,112]. This involves reconciling conflicting values, privacy norms, and cultural expectations, which constitute a social level of uncertainty. The “Collingridge dilemma” underscores the need to embed these considerations at the design stage to avoid irreversible lock-in [34]. LLMs, with their capacity for knowledge integration and contextual value reasoning, are being leveraged to construct agents with ethical alignment mechanisms [34,35,111,112]. As shown in Table 14, these agents explicitly model ethical preferences and social norms to manage value-laden uncertainty in scenarios involving multi-agent conflicts or moral trade-offs. Their role is to introduce a normative constraint layer that reduces the arbitrariness (or entropy) of decisions in socially sensitive contexts, thereby enhancing transparency, trust, and the system’s overall social entropy resilience.

**Table 14:** Representative Studies on Ethically Aligned Social Agents.

Ref.	Scenario	Role of LLM	Ethical Modeling Approach	Contributions	Limitations
[35]	AD ethical dilemmas	Core ethical decision-maker	Choice experiments with implicit value modeling; Logit and decision tree interpretation	Empirically decoded LLM moral preferences	Offline scenarios only
[34]	Human–machine symbiotic driving	Ethical and emotional alignment core	Emotion computing + value alignment + governance	Unified ethics, emotion, and governance	Theoretical framework
[111]	Traffic policy analysis	Social value analyzer	Legislative text analysis with LLM + XAI	Extended ethical analysis to policy formation	Single-country data
[112]	AD	Social norm–aware decision enhancer	Social behavior modeling + LLM reasoning	Enabled context-aware norm compliance	Simulation-based validation

#### 4.5.5. Summary and Analysis of Autonomous Agent Integration

**Figure 11:**
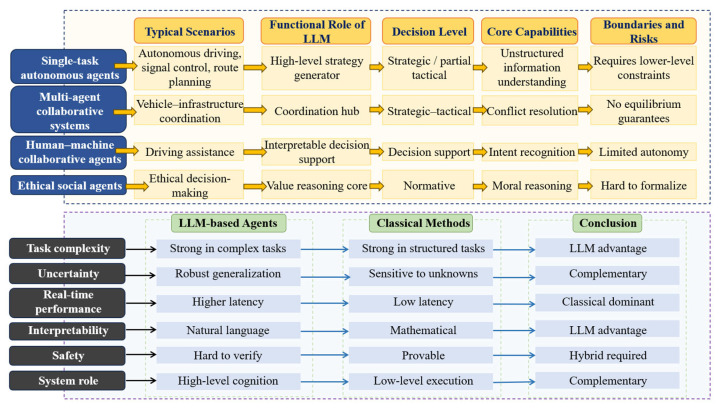
Scenarios, System Roles, and Capability Boundaries of LLMs at Autonomous Agent.

As synthesized in Figure 11, the research at the autonomous agent layer increasingly conceptualizes LLMs not as opaque tools but as centralized entropy processors and cognitive intermediaries. Their system role (varying from executor to mediator to arbitrator) directly determines their impact on the information dynamics and uncertainty profile of the traffic system. From an applicability perspective, LLM-driven agents demonstrate superior capability in scenarios characterized by high semantic, strategic, and social entropy, where objectives are complex and uncertainty is multifaceted. However, they are inherently limited in domains requiring ultra-low latency, deterministic safety guarantees, and verifiable convergence—areas where classical optimization and control methods maintain an advantage due to their lower computational and decisional entropy. Thus, the effective integration of LLMs hinges on a principled allocation of roles based on the type and magnitude of entropy each subsystem is designed to regulate.

## 5. Fundamental Limitations and Failure Modes of LLM-Enabled Traffic Systems

### 5.1. Limitations and Failure Mode Analysis

Although LLMs have substantially expanded the representational and reasoning capabilities of intelligent traffic systems, their limitations are not merely empirical or engineering-related, but structural and information-theoretic in nature. These limitations arise from a fundamental mismatch between the information (entropy) structures intrinsic to traffic systems and the probabilistic generative mechanisms of LLMs, as outlined in Section 3.5.

The structural tension identified in this paper does not refer to implementation difficulty but to a deep incompatibility between information-processing paradigms. Traffic systems are embedded in continuous, closed-loop physical environments governed by strong causal constraints, strict safety requirements, and multi-scale uncertainty propagation. Decision errors in such systems do not remain local but are amplified through feedback dynamics over time, making real-time predictability and risk bounding paramount.

By contrast, LLMs operate through discrete, autoregressive probabilistic inference over symbolic sequences. Their uncertainty management is primarily statistical and correlational, shaped by sequence-level entropy minimization objectives. While this enables efficient semantic compression, it does not provide intrinsic mechanisms for representing continuous state evolution, intervention-driven causal effects, or bounded risk under safety-critical, low-latency control loops. This mismatch manifests concretely as disparities in representation (discrete tokens vs. continuous metrics), uncertainty typology (semantic/epistemic vs. physical/aleatoric), and temporal behavior (stochastic latency vs. deterministic deadlines).

**Figure 12:**
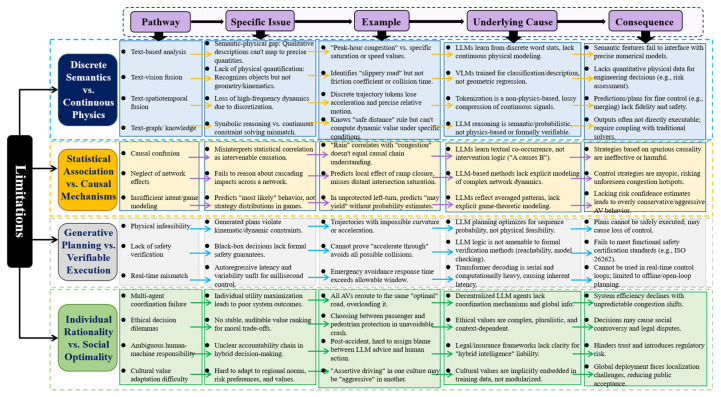
Capability bottlenecks and applicability boundaries of LLMs.

Consequently, even semantically coherent decisions from an LLM may induce unacceptable system-level risk when deployed in physical traffic loops. This tension cannot be resolved by scaling models or data but necessitates hybrid architectures with intermediate physical modeling and verification layers. Based on our four-level integration framework, Figure 12 summarizes the resulting capability bottlenecks, and Table 15 analyzes specific failure modes through this entropy-mismatch lens. The following subsections detail these limitations per integration level.

#### 5.1.1. Entropy Mismatch Between Discrete Semantic Spaces and Continuous Physical States

At the representation integration layer, a fundamental limitation arises from an inherent entropy mismatch between discrete semantic representations and continuous physical state spaces. Large language models operate by learning probability distributions over discrete tokens, whereas traffic systems evolve in continuous space governed by physical laws, kinematic constraints, and conservation principles [2,3,4,19,20,38,39,40,41,46,47,48,49]. This representational asymmetry creates a structural tension: while LLMs are highly effective at reducing semantic uncertainty, they lack mechanisms to preserve, reconstruct, or bound physically grounded state entropy.

This mismatch manifests consistently across different representation integration pathways. In pure text-based analysis, LLMs excel at compressing high-entropy, unstructured descriptions, such as accident reports or incident narratives, into coherent semantic summaries. However, this compression is inherently lossy with respect to physical entropy. Precise numerical quantities, including vehicle speed, density, headway, or impact geometry, cannot be reliably reconstructed from linguistic representations alone [36,44,45]. As a result, reductions in semantic entropy occur independently of, and often at the expense of, physical-state fidelity.

In text–visual fusion settings, multimodal LLMs primarily extract categorical labels, relational semantics, and scene-level descriptions. While such representations significantly reduce semantic ambiguity, metric uncertainty remains largely unresolved, limiting applicability in safety-critical perception tasks that require precise distance, velocity, or time-to-collision estimates [2,4,20,46,47]. The entropy reduction achieved here is therefore semantic rather than physical, reflecting abstraction rather than state estimation.

A similar pattern appears in text–spatiotemporal fusion. Tokenization and discretization of temporal sequences inevitably filter out high-frequency spatial and temporal variations [50,51,52,53,54,55,56,57,58,59,60,61]. This induces entropy collapse at fine scales, degrading performance in interaction-sensitive scenarios such as merging, car-following, or platooning, where small temporal deviations can have disproportionate physical consequences [59,60]. Once again, semantic regularities are preserved, while physically meaningful variability is attenuated or discarded.

In text–graph and knowledge integration, LLM-driven reasoning relies on semantic similarity and probabilistic association rather than strict constraint satisfaction. Logical consistency is evaluated in a symbolic or relational sense, but entropy-consistent inference under physical constraints, such as capacity limits or flow conservation, is difficult to guarantee [62,63,64,65,66,67,68,69]. Consequently, reductions in semantic uncertainty do not imply physically executable or dynamically consistent conclusions.

Recent literature provides concrete and reproducible evidence of this semantic–physical entropy mismatch at the representation layer. Zheng et al. [14] demonstrate that ChatGPT (GPT-3.5) can effectively automate accident report generation and extract high-level semantic attributes such as incident type and inferred contributing factors. However, they also document systematic failure cases in which the model hallucinates physically nonexistent entities or infers legal violations without sufficient evidentiary grounding. These errors do not stem from semantic incoherence, but from the lossy compression inherent in semantic entropy minimization, whereby probabilistic language modeling overwrites weakly specified or unobserved physical facts. This behavior constitutes a direct manifestation of representation-layer entropy mismatch.

A comparable asymmetry is observed in social-media-driven crash analysis. Jaradat et al. [102] employ fine-tuned LLMs on large-scale Twitter data to classify crash categories and extract descriptive attributes. While semantic uncertainty over narrative content is substantially reduced, the authors explicitly acknowledge that physically meaningful quantities, such as vehicle speed, spatial configuration, or impact dynamics, are neither recovered nor bounded. Reporting bias, incomplete descriptions, and demographic skew further decouple semantic representations from physical reality, leaving physical entropy unresolved despite strong linguistic performance.

Multimodal approaches amplify this pattern. Zhang et al. [40] propose the SeeUnsafe framework for video-based accident analysis using multimodal LLM agents. Although the system successfully transforms raw video into structured semantic descriptions, the authors report persistent ambiguity between near-miss and collision events under occlusion or single-view conditions. Fine-grained physical variables, including distance, velocity, and time-to-collision, remain inaccessible within the generated representations. Consequently, semantic entropy is sharply reduced, while physical entropy at the metric level remains weakly constrained.

Attempts to explicitly model uncertainty do not fully resolve this issue. de Zarzà et al. [41] introduce Bayesian uncertainty estimation alongside LLM and VLM reasoning, providing confidence measures over latent representations. However, the reported uncertainty reflects dispersion in abstract feature embeddings rather than bounded uncertainty over continuous traffic states. As such, the estimated entropy remains decoupled from executable physical constraints, illustrating that not all uncertainty quantification corresponds to physical entropy regulation.

Taken together, these studies consistently show that LLM-enabled representation integration excels at compressing heterogeneous textual and visual inputs into low-entropy semantic abstractions, while discarding or obscuring high-resolution physical state information. This loss is not an implementation artifact or data insufficiency, but a structural consequence of operating in discrete, symbol-centric representation spaces. It fundamentally constrains the role of LLMs at the representation layer: they function as powerful semantic compressors and interpreters, but cannot serve as complete physical perception modules. This insight clarifies why representation-level LLM integration, while valuable, must be coupled with domain-specific physical encoders, state estimators, or sensor-level models to bridge the semantic–physical entropy gap, thereby motivating the necessity of hybrid architectures emphasized throughout this review.

#### 5.1.2. From Correlation Entropy to Causal Uncertainty: Limits of Probabilistic Inference

At the reasoning and prediction integration layer, large language models (LLMs) demonstrate strong capability in modeling correlation entropy, namely statistical regularities and conditional likelihoods across heterogeneous spatiotemporal inputs [28,59,70,71,72,73,74,75]. By compressing high-dimensional historical observations into latent representations, LLMs effectively capture recurring traffic patterns and achieve competitive predictive accuracy. However, real-world traffic systems fundamentally require causal uncertainty modeling, where external interventions, feedback loops, and structural changes can abruptly alter the underlying data-generating process [2,11,12,13,76].

A critical limitation arises when LLM-based predictors implicitly treat frequently co-occurring patterns as stable causal relationships. Under distribution shifts or policy interventions, such correlation-based entropy minimization can lead to degraded or misleading predictions [13,51,52,53]. For example, TPLLM [49] introduces pretrained LLMs into traffic forecasting by embedding temporal sequences and spatial graphs before probabilistic extrapolation. While the framework demonstrates strong performance, particularly in small-sample scenarios, its inference mechanism remains observational: predictions are generated by extending historical correlations forward in time. When causal mechanisms change, such as new traffic regulations, infrastructure modifications, or extreme weather events, the model lacks the ability to identify or adapt to these shifts, potentially producing high-confidence yet incorrect outputs. In this case, correlation entropy is reduced, but causal uncertainty induced by unobserved interventions remains unresolved.

Similar limitations are observed in GPT4TFP [50], which employs spatio-temporal fusion via multi-head attention and leverages a frozen pretrained LLM to enhance predictive accuracy. Although the model achieves state-of-the-art performance on datasets such as NYCTaxi and CitiBike, its success relies on the strong stationarity assumption that historical traffic correlations persist. In practice, traffic systems are open and intervention-driven, where events such as pandemic controls or the opening of new transit lines fundamentally alter causal dependencies. GPT4TFP, like other correlation-centric approaches, cannot distinguish spurious correlations from causal relationships, nor can it quantify uncertainty stemming from unknown or changing mechanisms. Consequently, its uncertainty estimates reflect dispersion within observed correlations rather than causal (epistemic) uncertainty about unseen interventions or structural shifts.

Beyond temporal prediction, traffic networks also exhibit spatial and temporal uncertainty propagation, where localized disturbances cascade through route choices, signal coordination, and demand adaptation. Most LLM-based approaches lack explicit mechanisms to track or bound this propagated entropy at the network level [59,60,74]. Strada-LLM [58], for instance, enhances forecasting accuracy by incorporating graph structure and probabilistic modeling, treating uncertainty as variance over predicted distributions. However, the framework assumes relatively stable graph topology and does not differentiate uncertainty arising from stochastic variability versus that caused by missing or changing causal mechanisms. As a result, while probabilistic outputs improve robustness under mild distribution shifts, they do not resolve uncertainty induced by structural interventions or network reconfiguration.

Finally, multi-agent traffic systems involve strategic uncertainty, where outcomes depend on the interactive decisions of heterogeneous agents, including drivers, operators, and regulators. While LLMs tend to generate the most probable behavior or trajectory, they rarely represent full strategy distributions or equilibrium uncertainty. This limitation restricts their ability to reason about adversarial, non-cooperative, or game-theoretic interactions that are intrinsic to traffic systems [12,28,76]. As a result, current LLM-based traffic predictors remain largely confined to a descriptive statistical regime.

Taken together, these examples illustrate a fundamental gap between correlation entropy minimization and causal uncertainty modeling. While LLMs provide powerful tools for extracting and compressing statistical regularities, they do not, by design, infer causal structure or reason over interventions. This limitation is not an implementation artifact but a structural property of correlation-driven inference. Bridging this gap requires integrating LLMs with causal discovery, structural causal models, and counterfactual reasoning, enabling future traffic prediction systems to move beyond pattern continuation toward robust, interpretable, and intervention-aware decision support.

#### 5.1.3. Generative Planning vs. Entropy-Constrained Execution

At the planning and control integration layer, the conflict between generative entropy and executable certainty becomes explicit. LLM-based planners generate action sequences by sampling learned probability distributions, prioritizing semantic plausibility and contextual coherence rather than strict physical feasibility [22,24,25,26,81,82,83,84,85,86,87,88,89,90]. While this generative flexibility enables rich reasoning and adaptive planning, it fundamentally diverges from the requirements of safety-critical traffic control systems, which demand low-entropy, verifiable, and temporally bounded execution.

First, LLM-generated plans often violate implicit kinematic, dynamic, or operational constraints, because such constraints are not explicitly encoded in the entropy-minimization objectives underlying language modeling [22,24,83]. For example, LA-Light [24] leverages an LLM to reason over complex urban traffic scenarios, including emergency vehicle prioritization, roadblock incidents, and sensor outages, and to recommend signal phase decisions. Although the system demonstrates strong adaptability and explanatory clarity, the LLM does not directly execute signal control. Instead, it operates as a high-level reasoning and decision-support module, relying on traditional traffic signal control logic and auxiliary tools to ensure feasibility. This design choice implicitly acknowledges that semantic reasoning alone cannot guarantee constraint-consistent execution, and that physical and operational entropy must be regulated by non-generative control mechanisms.

Second, safety-critical traffic systems require verifiable entropy bounds on control outcomes, emphasizing worst-case guarantees rather than expected performance. However, the internal uncertainty of LLMs remains opaque and difficult to reconcile with formal verification or safety analysis frameworks [25,86]. In LLM-based actor–critic traffic signal control such as GPTTC [26], language models are embedded within a dual-agent architecture to improve adaptability and performance metrics such as delay and queue length. While empirical results show performance gains over classical controllers in near-saturated conditions, all evaluations are conducted in simulation environments under constrained scenarios. The LLM-driven policy operates within a reinforcement learning loop rather than providing independently verifiable guarantees, illustrating that probabilistic generative reasoning improves average performance but does not furnish certifiable safety bounds.

Third, real-time traffic control imposes stringent latency and determinism requirements. Decisions such as collision avoidance, signal phase switching, or lane-change authorization often require responses within tens of milliseconds. Autoregressive LLM inference, however, introduces stochastic latency due to sequence length, decoding strategies, and hardware scheduling [25,86,87]. This latency variability is not merely an implementation artifact but a source of physical-state and causal uncertainty (temporal entropy) that accumulates in closed-loop systems. In HighwayLLM [85], for instance, LLM modules are explicitly restricted to high-level trajectory planning or safety validation, while low-level control remains governed by reinforcement learning agents. The reported inference times on the order of several seconds far exceed human reaction times and are acknowledged by the authors as unsuitable for real-time autonomous driving, reinforcing the necessity of strictly separating generative reasoning from time-critical execution.

Taken together, these representative systems illustrate a consistent architectural pattern: even when LLMs are successfully integrated into planning and control pipelines, they are deliberately confined to high-level, advisory, or supervisory roles, while execution is delegated to classical controllers, rule-based logic, or reinforcement learning modules with bounded latency and constrained entropy. This separation is not accidental but reflects a fundamental incompatibility between generative entropy and entropy-constrained execution. The limitation therefore does not stem from insufficient training data or model scale, but from the intrinsic mismatch between probabilistic sequence generation and the deterministic, verifiable requirements of safety-critical traffic control. Consequently, effective deployment of LLMs at this layer necessitates hybrid architectures in which generative planning is tightly coupled with low-entropy execution and formal verification mechanisms.

#### 5.1.4. Individual Rationality, Social Entropy, and System-Level Instability

At the autonomous agent layer, the limitations of LLM-enabled systems extend beyond individual decision quality to system-level entropy amplification, where locally rational behaviors collectively induce instability, inefficiency, or unintended emergent dynamics [23,34,35,43,106,110,111,112]. LLM-driven agents typically optimize local expected utility based on internally learned probabilistic beliefs and contextual reasoning. However, these beliefs are not inherently coordinated across agents, nor are they grounded in explicit equilibrium or global consistency constraints.

This phenomenon is clearly illustrated in agentic route choice modeling. In LLMTraveler [43], LLM-based agents successfully reproduce human-like day-to-day route switching behaviors and generate plausible natural-language explanations for individual decisions. While the framework demonstrates strong behavioral realism at both individual and aggregate levels, it explicitly focuses on single-agent rationality under partial memory and experience. No mechanism is introduced to enforce network-level coordination or to analyze equilibrium convergence under large-scale deployment. As a result, when such agents are scaled across many origin–destination pairs, synchronized exploration–exploitation dynamics may emerge, potentially amplifying demand fluctuations and inducing secondary congestion—an instance of social entropy amplification arising from uncoordinated individual entropy minimization.

Similar patterns appear in multi-agent traffic control. Chen and Meng [90] integrate LLMs into a MARL framework to enhance coordination among signal control agents, achieving improved efficiency and convergence speed in multi-intersection scenarios. However, stability is assessed empirically through simulation rather than guaranteed analytically. The introduction of LLM reasoning improves local collaboration heuristics but does not provide formal guarantees on long-horizon stability, robustness under demand surges, or resilience to adversarial perturbations. Consequently, while average performance improves, system-level entropy remains unbounded, particularly as the number of interacting agents grows.

More explicitly, the CCMA framework [107] exposes the tension between individual optimization and collective stability. CCMA employs a hierarchical design in which reinforcement learning governs individual-level behavior, while LLMs coordinate regional and global interactions among autonomous vehicles during merging. Experimental results demonstrate high success rates and efficiency under low and medium traffic densities. However, the authors explicitly acknowledge performance degradation in high-density scenarios, attributing it to limitations in communication protocols and coordination mechanisms. This admission highlights a structural issue: even with LLM-mediated cooperation, local rationality does not automatically translate into global stability, and increasing interaction density amplifies social entropy rather than suppressing it.

Beyond operational performance, ethical and social decision-making further compounds system-level uncertainty. LLMs lack stable, auditable mechanisms to manage such value entropy across contexts [34,35,112]. Dong et al. [34] analyze LLM-integrated human–machine symbiosis in intelligent driving, showing that affective reasoning and persuasive interaction introduce normative and institutional entropy. Value trade-offs, such as safety versus autonomy, assistance versus control, cannot be reduced to a single scalar objective. Moreover, responsibility attribution between human drivers, automated systems, and developers remains ambiguous. LLMs lack stable, auditable mechanisms to manage such value entropy across contexts, leading to legal and governance uncertainties that scale with system adoption.

Taken together, these representative studies reveal a consistent pattern: LLM-enabled agents often achieve impressive individual-level rationality, adaptability, and interpretability, yet system-level coordination, stability, and accountability remain unresolved. The resulting gap between individual entropy minimization and collective entropy regulation constitutes a fundamental limitation of current LLM-based multi-agent traffic systems. This limitation is not an implementation flaw but a structural challenge, underscoring the need for explicit coordination protocols, equilibrium-aware learning, and governance mechanisms to prevent social entropy amplification in large-scale deployments.

### 5.2. Boundaries of LLM Irreplaceability and Hybrid Intelligence Architectures

**Figure 13:**
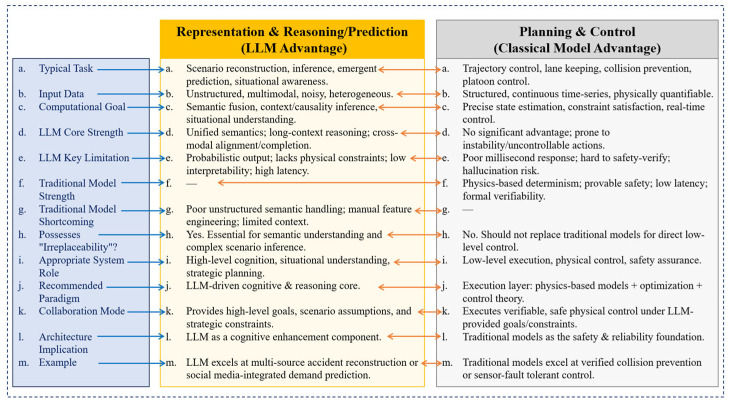
Comparative Analysis of LLM vs. Traditional Model Capabilities Across Transportation Task Levels. The yellow panel highlights task layers where LLMs exhibit relative advantages in representation and reasoning/prediction, while the gray panel denotes dominated by classical planning and control models. The light blue column lists comparison dimensions shared by both paradigms. Solid blue arrows indicate alignment between task attributes and the corresponding strengths or limitations of each modeling approach. Bidirectional orange arrows emphasize conceptual contrasts and information exchange between LLM-based cognitive layers and physics-based control layers. The outer blue dashed boundary represents an integrated system architecture, indicating that LLMs and traditional models are complementary components rather than substitutes. Dashed elements denote conceptual grouping instead of strict implementation boundaries.

Despite the intrinsic limitations, LLMs continue to represent a profoundly promising avenue for advancing traffic intelligence, primarily due to their unique capacity for semantic entropy management [1,5,15]. This necessitates a fundamental inquiry: from an information-theoretical perspective, in which traffic tasks LLMs demonstrate irreplaceable advantages, and in which scenarios do classical models retain superiority? The answer, as synthesized in Figure 13, hinges on the specific type and magnitude of entropy a given task demands to be regulated.

At the representation integration and reasoning–prediction levels, the irreplaceable value of LLMs resides in their ability to perform unified semantic compression and contextual entropy reduction across unstructured, multimodal data streams [2,3,4,12,13,19,20,38,39,40,41,46,47,48,49,67,73,74]. Their core strength is constructing cross-modal informational bridges and modeling long-range contextual dependencies, effectively transforming high-entropy, heterogeneous inputs into coherent, low-entropy semantic representations, which is inherently a challenge for classical, modality-specific models [2,3,4,19,20,67].

Conversely, at the planning and control level, tasks are characterized by the imperative for ultra-low physical, temporal, and logical entropy. In domains requiring high-precision kinematic modeling, formally verifiable safety guarantees, and millisecond-level deterministic response, the probabilistic generative entropy and high-latency epistemic uncertainty of LLMs render them currently unsuitable to surpass classical control and optimization models [22,24,25,81,83,86,87]. The fundamental misalignment stems from the conflict between the LLMs’ statistical, high-entropy decision processes and the rigid requirements for physical determinism and temporal reliability [25,26,27,86].

Hence, it directs the future toward hybrid intelligence architectures, conceived as layered entropy-regulation systems. In such frameworks, LLMs would serve as the high-level cognitive core, specializing in managing strategic, semantic, and causal uncertainty entropy. They would generate abstract plans, infer latent constraints, and provide contextual guidance. In parallel, classical models would act as the low-level actuation layer, entrusted with executing physical actions under minimal temporal and operational entropy, ensuring safety, precision, and real-time performance [21,22,24,25,81,83,90,108].

The pivotal research challenge, therefore, transcends mere component integration. It lies in the design of robust, entropy-aware interfaces and coordination mechanisms. These interfaces must efficiently translate high-level, semantic directives from the LLM into low-entropy, executable commands for the classical layer, while simultaneously feeding back precise physical state information to ground the LLM’s reasoning. Successfully engineering this bi-directional information flow with managed entropy loss is critical for realizing reliable, efficient, and socially aligned traffic intelligence systems [1,107].

## 6. Research on Future Agendas for LLM-Based Traffic Applications

**Figure 14:**
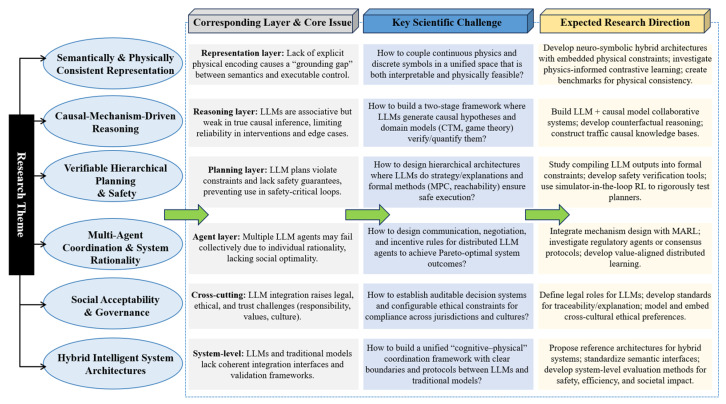
Future Research Agenda for LLM-Based Traffic Applications.

The preceding analysis reveals that the integration of LLMs into traffic systems is fundamentally constrained not by model capacity alone, but by how uncertainty, information, and entropy are represented, transformed, and controlled across system layers. As traffic systems are inherently open, stochastic, and safety-critical information-processing systems, future progress cannot rely solely on scaling model size or computational resources. Instead, it requires a restructuring of LLM-centric research agendas around entropy management, probabilistic consistency, and information-theoretic alignment with physical and social constraints. The key challenge is not to maximize predictive accuracy, but to regulate how uncertainty propagates across semantic, decision, and execution layers. Figure 14 illustrates major future research directions from a system-level viewpoint. To complement this application-oriented visualization, Table 16 provides a structured summary of future research agendas explicitly framed in terms of entropy, information theory, and probability theory. Table 16 highlights that future research directions are fundamentally questions of entropy regulation rather than isolated algorithmic improvements.

## 7. Conclusions

This survey has examined the integration of LLMs into intelligent traffic systems through an information-theoretic lens. By organizing existing studies into four progressive integration levels (representation, reasoning and prediction, planning and control, and autonomous agents), we demonstrate that the opportunities and limitations of LLM-based traffic intelligence are governed by how entropy and uncertainty are processed across system layers.

At the representation level, LLMs exhibit strong capabilities in semantic entropy reduction by unifying heterogeneous data modalities, yet they face intrinsic limits when discrete language abstractions are mapped onto continuous physical states. At the reasoning and prediction level, LLMs improve contextual inference under uncertainty but remain constrained by their reliance on statistical correlation rather than explicit causal entropy modeling. At the planning and control level, the probabilistic and generative nature of LLMs conflicts with the low-entropy, verifiable execution demanded by safety-critical traffic operations. At the autonomous agent level, individual uncertainty-aware reasoning does not necessarily translate into system-level entropy minimization, raising challenges in coordination, fairness, and ethical accountability.

Crucially, these limitations should not be interpreted as temporary technical deficiencies. Instead, they reflect structural mismatches between probabilistic language modeling and the physics-governed, tightly constrained nature of traffic systems. LLMs are optimized for high-uncertainty semantic environments, whereas traffic control requires aggressive uncertainty suppression and predictable state evolution.

Accordingly, this review argues that the future of intelligent traffic systems lies in entropy-aware hybrid intelligence architectures, where LLMs serve as high-level semantic reasoners and uncertainty-structuring components, while classical physics-based, optimization-driven, and control-theoretic models ensure deterministic execution, safety guarantees, and real-time stability. The core scientific challenge is not replacing traditional models but designing principled mechanisms that regulate information and entropy flow across cognitive and physical layers.

Looking forward, we identify key research directions centered on causality-aware uncertainty modeling, entropy-constrained planning and verification, multi-agent entropy coordination, and uncertainty-calibrated human–machine interaction. Advancing these directions will enable LLMs to evolve from powerful probabilistic pattern learners into reliable components of safety-critical, large-scale traffic information systems.

Finally, this review acknowledges its limitations, including incomplete coverage of industrial deployments, limited discussion of evaluation and reproducibility challenges, and the exclusion of non-English literature. Future surveys should pursue longitudinal and comparative analyses across complex systems domains, with particular emphasis on entropy, uncertainty governance, and accountability in AI-driven transportation systems.

## Figures and Tables

**Table 1:** Representative Studies of LLM-Based Pure Text Analysis.

Ref.	Traffic Task	LLM’s Core Role	Text Processing & Output	Key Contribution
[44]	Crash severity analysis	Embedding generator & latent pattern discoverer	Process: Crash narratives, deep semantic representations. Output: Risk clusters & high-frequency themes.	Automatically identifies latent crash factors from text without predefined variables.
[45]	Grievance identification	Representation learner & classifier	Process: Social media text, Transformer embeddings. Output: Multi-label grievance vectors & categories.	Converts public text into structured semantics for governance, no handcrafted rules needed.
[36]	Travel mode prediction	Personalized feature extractor	Process: Travel records (as NL), MLM embeddings. Output: Semantic vectors of personal preferences & context.	Models travel via semantic abstraction, capturing individual preferences.

**Table 15:** Failure Modes of LLM-Enabled Traffic Systems.

Integration Layer	Core Entropy Mismatch	Failure Mode	Practical Consequence
Representation	Semantic entropy vs. physical entropy	Lossy physical information compression	Reduced precision and safety margin
Reasoning & Prediction	Correlation entropy vs. causal uncertainty	Poor intervention generalization	Fragile predictions under policy change
Planning & Control	Generative entropy vs. executable certainty	Constraint violations, unverifiable plans	Safety and real-time risks
Autonomous Agents	Individual entropy minimization vs. social entropy	Coordination failure, instability	System-level inefficiency

**Table 16:** Entropy-Oriented Future Research Agenda for LLM-Enabled Traffic Systems.

System Layer	Entropy/Uncertainty Source	Core Research Question (Entropy View)	LLM Role	Required Methodological Advances
Representation Integration	High-dimensional multimodal noise; semantic ambiguity	How can semantic entropy be minimized without discarding physically relevant information?	Semantic compression and mutual information preservation	Information bottleneck methods; entropy-regularized representation learning; uncertainty-aware multimodal fusion
Reasoning & Prediction	Stochastic demand, human behavior, network interactions	How can probabilistic inference distinguish correlation entropy from causal uncertainty?	Probabilistic reasoning and hypothesis generation	Causal entropy modeling; Bayesian LLM hybrids; intervention-aware uncertainty estimation
Planning & Control	Execution uncertainty; physical constraints; safety risks	How can generative uncertainty be constrained to meet low-entropy execution requirements?	High-level plan proposal under uncertainty	Entropy-constrained planning; formal verification interfaces; probabilistic-to-deterministic projection mechanisms
Autonomous Agents	Strategic uncertainty; multi-agent interaction entropy	How does individual uncertainty aggregation affect system-level entropy and stability?	Strategy modeling and negotiation	Game-theoretic entropy analysis; equilibrium uncertainty modeling; social welfare–entropy tradeoff mechanisms
Human–Machine Interaction	Cognitive uncertainty; trust and interpretability gaps	How can uncertainty be communicated and calibrated between humans and LLM agents?	Decision explanation and risk communication	Uncertainty calibration; information-theoretic interpretability; entropy-aware human-in-the-loop design

## Data Availability

Data accessibility does not apply to this article, as no new data was created in this study.

## Abbreviations

The following abbreviations are used in this manuscript:

ABM	Agent-Based Modeling
AC	Actor-Critic
ACP	Artificial societies, Computational experiments, Parallel execution
AD	Autonomous Driving
AV	Autonomous Vehicle
BERT	Bidirectional Encoder Representations from Transformers
CAV	Connected and Autonomous Vehicle
CNNs	Convolutional Neural Networks
CoT	Chain-of-Thought
DB	Database
DL	Deep Learning
EIVM	Externally Integrated Vision Modality Model
EV	Electric Vehicle
GAT	Graph Attention Network
GCA	Generally Capable Agent
GCN	Graph Convolutional Network
GNNs	Graph Neural Networks
GPS	Global Positioning System
GPT	Generative Pre-trained Transformer
HDV	Human-Driven Vehicle
HMI	Human–Machine Interaction
IoT	Internet of Things
KG	Knowledge Graph
LiDAR	Light Detection and Ranging
LLaMA	Large Language Model Meta AI
LLMs	Large Language Models
LoRA	Low-Rank Adaptation
MAE	Mean Absolute Error
MAPPO	Multi-Agent Proximal Policy Optimization
MARL	Multi-Agent Reinforcement Learning
MoE	Mixture-of-Experts
MPC	Model Predictive Control
MLM	Masked Language Model
MTD	Multimodal Traffic Dataset
MTL	Metric Temporal Logic
NL	Natural Language
NLP	Natural Language Processing
OD	Origin-Destination
OOD	Out-Of-Distribution
PCA	Principal Component Analysis
PID	Proportional-Integral-Derivative
QA	Question Answering
RAG	Retrieval-Augmented Generation
Rep.	Representation
RL	Reinforcement Learning
RMSE	Root Mean Square Error
RNNs	Recurrent Neural Networks
SQL	Structured Query Language
ST-LLM	Spatio-Temporal Large Language Model
SUMO	Simulation of Urban Mobility
TSP	Traveling Salesman Problem
V2X	Vehicle-to-Everything
VAEs	Variational Autoencoders
VICS	Vehicle Intention-Based Control Signals
VLMs	Vision-Language Models
VQA	Visual Question Answering
XAI	Explainable Artificial Intelligence
